# Multi-marker metabarcoding of coral skeletons reveals a rich microbiome and diverse evolutionary origins of endolithic algae

**DOI:** 10.1038/srep31508

**Published:** 2016-08-22

**Authors:** Vanessa Rossetto Marcelino, Heroen Verbruggen

**Affiliations:** 1 School of Biosciences, University of Melbourne, VIC 3010, Australia

## Abstract

Bacteria, fungi and green algae are common inhabitants of coral skeletons. Their diversity is poorly characterized because they are difficult to identify with microscopy or environmental sequencing, as common metabarcoding markers have low phylogenetic resolution and miss a large portion of the biodiversity. We used a cost-effective protocol and a combination of markers (*tuf*A, 16S rDNA, 18S rDNA and 23S rDNA) to characterize the microbiome of 132 coral skeleton samples. We identified a wide range of prokaryotic and eukaryotic organisms, many never reported in corals before. We additionally investigated the phylogenetic diversity of the green algae—the most abundant eukaryotic member of this community, for which previous literature recognizes only a handful of endolithic species. We found more than 120 taxonomic units (near species level), including six family-level lineages mostly new to science. The results suggest that the existence of lineages with an endolithic lifestyle predates the existence of modern scleractinian corals by *ca*. 250my, and that this particular niche was independently invaded by over 20 lineages in green algae evolution. These results highlight the potential of the multi-marker approach to assist in species discovery and, when combined with a phylogenetic framework, clarify the evolutionary origins of host-microbiota associations.

Corals harbour a diverse microbial community that is vital for their health and resilience^1^,^2^. The skeletons of stony corals are populated by endolithic (limestone-boring) bacteria, fungi and a conspicuous layer of green algae^3^,^4^. These organisms are protected from the external environment but endure very low levels of light and extreme daily fluctuations of pH and oxygen levels^5^. The endolithic habitat therefore contains a specialized microbial community, and very little is known about when or how many times the association between corals and these organisms has evolved.

Endolithic algae constitute a major component of the endolithic microbiome in terms of abundance and ecological roles. They are the principal microbial agent of reef erosion^6^,^7^ and increase coral decalcification under elevated acidity and temperature^8^. These algae also protect corals from high-light stress^9^ and provide them with an alternative source of energy during bleaching events^10^. The balance of benefits and drawbacks that these algae convey to corals is unclear and likely depends on the interplay between different algal lineages and other microorganisms in the coral holobiont. However, these organisms are mostly uncharacterized.

*Ostreobium* (Ulvophyceae, Chlorophyta) is considered to be the most abundant endolithic algal genus in marine habitats and has three described species (although there are some inconsistencies in literature). It is a siphonous alga, meaning that its whole body consists of a single, branched, multinucleate cell^11^. This simple architecture evidently puts strong limits on the number of morphological characters available to distinguish different species^12^. A pilot study on the *rbc*L gene diversity in *Ostreobium* found seven genotypes, indicating that the number of species is higher than the taxonomic literature suggests^13^. Several other micro-eukaryotic groups in coral skeletons possibly also have uncharted cryptic diversity (e.g. fungi), demanding approaches to study their biodiversity that do not rely on morphological identification.

Metabarcoding allows for in-depth microbial composition assessments directly from environmental samples^14^. This approach has led to the discovery of an enormous number of microorganisms never isolated or cultured before. Those organisms–coined microbial dark matter–account for the majority of microbial diversity^15^,^16^. There is an even darker category of organismal matter though–those that are undetectable with commonly used metabarcoding methods. The fraction of the biodiversity captured by metabarcoding surveys depends on the markers and primers used, so organisms that are not amplified with the standard methods go undetected even if they are common and play important roles in the ecosystem.

Endolithic algae illustrate how common and important organisms can be virtually ignored in metabarcoding surveys that use a single standard marker. Although the coral microbiome is relatively well studied, researchers using the 16S rDNA are generally interested in the bacterial community and often discard chloroplast reads (e.g^17^). Eukaryotic surveys based on 18S rDNA possibly underestimate algal diversity because they can be biased towards heterotrophs^18^. The use of group-specific markers with higher phylogenetic resolution improves the recognition of closely related organisms (e.g. cryptic species) and allows phylogeny-based evolutionary inferences. Comprehensively surveying all microbial diveristy would require sequencing both universal and high-resolution markers. Such approaches would facilitate capturing an eclectic range of co-occurring microorganisms and simultaneously getting a deeper understaing of particular taxa of interest.

Multi-marker strategies have not been extensively used for two main reasons. First, library preparation becomes expensive for multiple markers and there are no automated protocols available to study the less commonly used markers. Second, non-standard markers have relatively poor reference datasets compared to what is available for 16S and 18S rDNAs, hence the classification of the retrieved sequences by conventional methods (e.g. RDP classifier^19^ or BLAST^20^) is problematic^21^. In such cases, operational taxonomic units (OTUs) are better classified using a phylogenetic framework, which has the added advantage of providing an historical evolutionary perspective.

We investigated the diversity of the prokaryotic and eukaryotic microbiome in coral skeletons using a cost-effective multi-marker metabarcoding protocol and evaluated the benefits of different markers. Because of their abundance and importance, we focused on the biodiversity and evolution of the green algae in the endolithic community. Using phylogenetic methods, we inferred when and how many times the association with a coral-endolithic habitat emerged in the evolutionary history of green algae.

## Results

### Cost-effective biodiversity assessment

We sequenced 132 coral skeleton samples collected in Australia and Papua New Guinea from a wide variety of habitats and coral genera (Supplementary Materials). In order to obtain information from eukaryotic and prokaryotic members of the microbiome, we used four metabarcoding markers: the 16S rDNA^22^, the 18S rDNA^23^, a fragment of the 23S rDNA that targets algal chloroplasts^24^ and a fragment of the elongation factor Tu (*tuf*A) gene, a DNA barcode recommended and commonly used for green algae due to its ability to distinguish between closely related species^25^.

**Figure 1:**
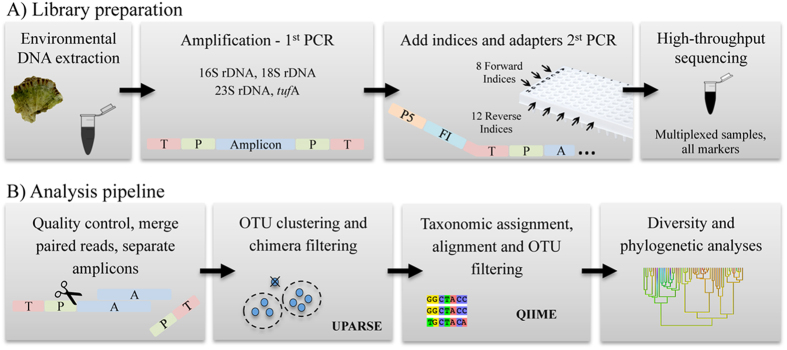
The multi-marker metabarcoding approach. The library preparation (**A**) consists of a 2-step PCR amplification: the first PCR amplifies the target markers and the second PCR adds the indices and the Illumina adapters (T = tail, P = amplicon-specific primer, P5 = Illumina adapter, FI = forward index, A = amplicon). The amplicons are then purified with magnetic beads, quantified and pooled together to be sequenced in an Illumina’s MiSeq platform. The sequence reads of the 4 markers are teased apart in the analysis pipeline (**B**) based on primers sequences, and go through a series of quality control steps (including pipelines available in QIIME^28^), OTU clustering (using UPARSE^46^), alignment and classification.

We used a cost-effective multi-marker metabarcoding approach that uses a two-step PCR protocol to amplify the markers and prepare the Illumina library, replacing a commonly used kit (Nextera Index kit) with custom made oligos, reducing the indexing costs by 60 times (AUD $3.28 *vs*. $0.05 per sample, Supplementary Materials). When compared to the Earth Microbiome Project protocol^26^, this approach has the advantage of requiring only 20 indexing oligos (Fig. 1A), plus first PCR primers, instead of 384 (plus 4 reverse primers) for 96 samples and 4 markers. Using home-made magnetic beads as described in Rohland & Reich^27^ decimated the costs of cleaning PCR products. We obtained, on average, 161,364 sequences per sample (comprising 4 amplicons each). Following removal of low quality sequences, 14,131,986 sequences were retained, of which 2,603,384 were in the 16S rDNA dataset, 4,102,867 in the 18S rDNA, 4,607,505 in the 23S rDNA and 2,818,230 sequences in the *tuf*A dataset. These sequences were deposited in NCBI’s Sequence Read Archive (SRA) under the accession ID SRP073961.

### Multi-marker microbiome characterization

**Figure 2:**
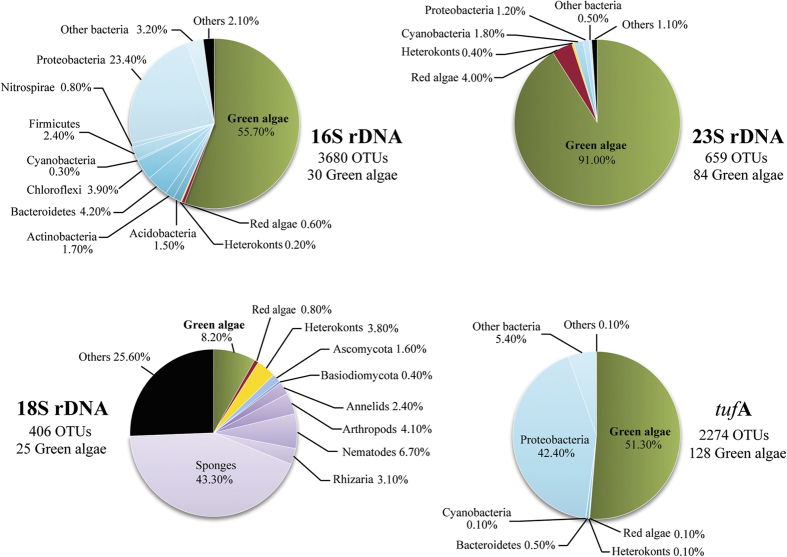
Pie charts indicating the relative abundance of sequence reads matching the main taxa assigned with RDP-classifier. Note that the relative abundances do not always reflect diversity, as indicated by the total and green algal number of Operation Taxonomic Units retrieved with each marker.

To analyse the overall microbiome diversity, we made an assessment of all OTUs classified via RDP classifier as implemented in QIIME^19^,^28^. Our results showed that the widely used 16S and 18S rDNA markers drastically underestimate green algae diversity. In total, 3,680 OTUs were found in the 16S rDNA survey, 406 in the 18S rDNA, 659 in the 23S rDNA and 2,274 in the *tuf*A dataset. Green algal reads were the most abundant in all but 18S rDNA dataset (Fig. 2). Note that the living coral tissue was removed prior to DNA isolation, therefore the high relative abundance of algal reads when compared to bacterial reflects the densely algae-populated skeletons. Besides *Ostreobium*, several other photosynthetic lineages were found including other green algae, red algae, brown algae and cyanobacteria, most of which are organisms or lineages not previously known to occur in coral skeletons.

Although green algae accounted for 55.7% of the 16S sequences, only 30 out of 3,680 OTUs were assigned to this group, and only 3 OTUs were classified beyond the rank of class (Ulvophyceae)–1 OTU with the closest match to *Bryopsis hypnoides* (confidence score = 0.6), and 2 OTUs classified as *Chlorodesmis fastigiata* (confidence score >0.7). Red algae and cyanobacteria composed 0.8% and 0.3% of 16S rDNA reads, respectively. The alphaproteobacterial order *Rhizobiales*, thought to occur exclusively within the coral tissue^29^, was found in considerable abundance (2.3%) among our reads (see also^30^), although the possibility of contamination from coral tissue cannot be entirely ruled out. Green sulphur bacteria (phylum Chlorobi) composed 0.1% of the reads in our 16S rDNA dataset.

The 18S rDNA metabarcode captured mostly endolithic sponges (42.3%), but also occasional nematodes, arthropods, annelids and fungi (Fig. 2). Sixteen OTUs (1.9% of relative abundance) were assigned to *Labyrinthula* (confidence score >0.7), a heterokont genus known to infect algae^31^. Green algae composed 8.2% of the sequence reads, comprising 25 OTUs. 7 OTUs were classified to genus rank (confidence score > 0.7): 4 *Cladophora*, 1 *Pseudulvella* and 2 *Phaeophila*. None of the reads corresponded to *Ostreobium* despite it clearly being abundant in the samples. Brown algae composed 0.5% of the reads, with 11 OTUs, mostly *Ectocarpales*.

In the 23S rDNA dataset, the vast majority of reads were assigned to green algae (91%—84 OTUs), red algae (4%) and bacteria (3.5%). Cyanobacteria were present in low relative abundance but were diverse. Of the 92 cyanobacterial OTUs, 5 matched *Acaryochloris marina* with high confidence scores (0.87–1.00).

The *tuf*A reads were composed of green algae (51.3%), bacteria (48.4%) and a small fraction of red algae and heterokonts (Fig. 2). This marker retrieved the highest number of green algal OTUs (128) of which 53 were classified as *Ostreobium*. Three other algal genera were found with high confidence scores (0.85–1.00): 2 OTUs in *Halimeda*, 2 *Phaeophila* and 1 *Ulvella*. Other OTUs were only classified at higher taxonomic ranks or with lower confidence scores.

### Phylogenetic diversity and evolution of endolithic green algae

**Figure 3:**
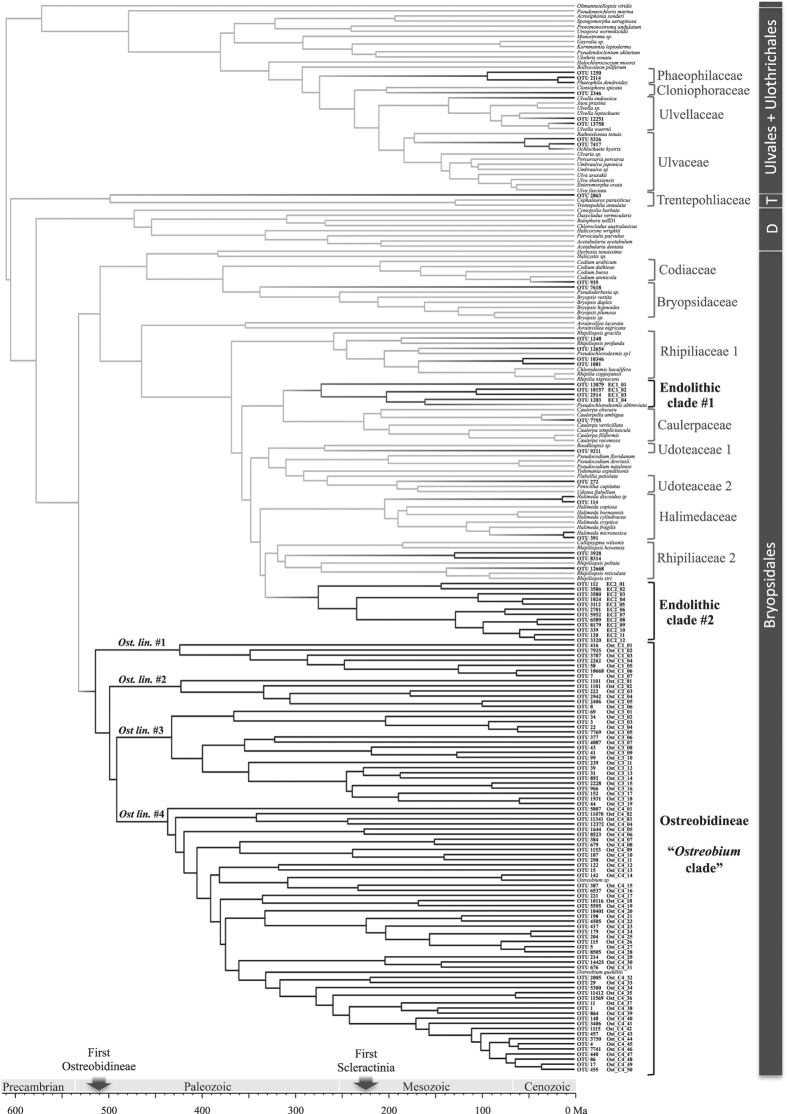
Maximum Likelihood tree of green algae (4833 bp alignment) including the OTUs retrieved with the t*uf*A metabarcode. The green algal families where OTUs were found are indicated in square brackets. The OTUs composing the endolithic clades were given an identifier for reference in future biodiversity screenings using the *tuf*A marker. The ancestral states reconstruction of the endolithic nature is plotted along the tree and indicate the probability of the ancestral lineage being coral-endolithic (black) or not (grey).

We studied the diversity of green algae in more detail by building phylogenetic trees from the retrieved eukaryotic OTUs (as classified by RDP) and available reference sequences (Fig. 3 and Supplementary Figs S1–S4). The use of a phylogenetic framework allowed identifying more green algal OTUs than the RDP classifier did. In the 16S data, 36 OTUs were green algae (*versus* 30 classified with RDP), 21 of which were in the *Ostreobium* clade (Supplementary Fig. S2). The phylogeny of the 18S OTUs confirmed the absence of *Ostreobium* reads in this dataset, and the presence of 5 OTUs in the *Cladophora* genus (Supplementary Fig. S3). The 23S rDNA dataset revealed 79 OTUs within core Chlorophyta, of which 61 were in the *Ostreobium* clade (Supplementary Fig. S4).

The *tuf*A gene has a better phylogenetic resolution than other markers^25^ and allowed us to perform a detailed analysis of phylogenetic diversity and evolution of the green algal OTUs (Fig. 3, Supplementary Fig. S1). We excluded 7 green algal *tuf*A OTUs that did not fall within the core Chlorophyta clade. Of the 121 remaining OTUs, one belonged to the Trebouxiophyceae and 120 were Ulvophyceae. Endolithic OTUs were found in 11 families in Ulvophyceae (plus Cladophoraceae in 18S rDNA dataset), some OTUs were distantly related to known algae while others were very similar or identical to known seaweeds never reported in coral skeletons (Fig. 3, bootstrap values in Supplementary Fig. S1). With the two previously published *Ostreobium tuf*A sequences, 82 OTUs formed a well-supported, early-branching clade that was further split into 4 subclades, also well supported. We discovered another two endolithic clades: one including *Pseudochlorodesmis* (4 OTUs) and a second one sister to the family Rhipiliaceae (12 OTUs). The ancestral reconstruction of the coral-endolithic nature in green algae indicated that this trait evolved more than 20 times independently (Fig. 3). The time-calibrated phylogeny also allowed comparing the age of families across different parts of the tree and extrapolating this age to the newly discovered lineages. It suggested that the endolithic algae found in this study represent at least six family-level lineages (four subclades of the *Ostreobium* clade and endolithic clades #1 and #2).

## Discussion

### Multi-marker view of coral skeleton microbiome

This study highlights how multi-marker approaches can enrich biodiversity surveys. Our results show that the commonly used 16S and 18S rDNA markers severely underestimate algal diversity, and that no metabarcode, in isolation, is sufficient to characterize complex microbiomes. The multi-marker data increase the range of microbial taxa recovered from the samples and yields massive savings when compared to traditional methods (Fig. 1).

The multi-marker method allows combining the qualities of each marker for more comprehensive biodiversity surveys (e.g.^32^). The 16S rDNA, for example, retrieved the highest number of OTUs and is convenient for cross-comparability with the vast number of studies focused on bacterial communities, but it underestimates algal diversity. Commonly used universal primers for highly conserved rRNA genes (16S and 18S) capture a wide range of microbial taxa at the expense of losing power to detect closely related species. The *tuf*A marker has a higher rate of evolution especially at third codon positions and yields many more green algal OTUs as well as a better-supported phylogeny (Supplementary Figs S1–S4). Further, some organisms are difficult or impossible to amplify with standard primer pairs, presumably due to substitutions at primer binding sites, but can be detected using the multi-marker approach. For instance, the 18S rDNA marker did not retrieve any OTU of the siphonous green algae (Bryopsidales), while 112 were obtained with *tuf*A. Nevertheless, the 18S rDNA was the only marker yielding OTUs of another order of green algae (Cladophorales; Supplementary Fig. S3). Like dinoflagellates, this green algal order possesses an unusual plastid configuration that prevents amplification with standard plastid markers^33^. We found 5 OTUs in the Cladophorales and this is the first record of their occurrence in coral skeletons.

Corals harbour a particular microbiome in their skeletons^29^,^30^. *Alphaproteobacteria* and *Gammaproteobacteria* were the predominant prokaryotic members, in agreement with some metabarcoding studies of coral skeletons^30^,^34^. A recent study indicates that green sulphur bacteria are prevalent in skeletons of *Isopora palifera*^35^. We found that these bacteria compose only a small fraction of the prokaryotic community in the corals analysed here (in agreement with one other study targeting endolithic bacteria^30^). We also found a diverse community of cyanobacteria, which was best characterized with the 23S rDNA marker. To our knowledge, this is the first record of *Acaryochloris marina* in skeletons of living corals. This cyanobacterium produces chlorophyll-*d* and is known to occupy niches depleted of visible light^36^. Many other cyanobacterial OTUs could not be classified at lower taxonomic ranks with the RDP classifier, but might reveal interesting groups specialized in the endolithic niche when analysed in a phylogenetic context.

### Highly diverse endolithic green algae

We found that the genus *Ostreobium*, previously thought to be composed of only three species, is a 500 million year-old complex comprising more than 80 taxonomic units at the near-species level (Fig. 3). The lineage is divided into four well-supported subclades with divergence times comparable to the family level in the seaweed lineages of the siphonous green algae (Fig. 3). A recent study in which reef rubble and coralline algae were sequenced also revealed a large *Ostreobium* diversity^37^.

Our results also revealed a large number of green algae outside the Ostreobidineae clade that were not known to occur in coral skeletons. One of the lineages–endolithic clade #2 in Fig. 3–is exclusively composed of endolithic algae never described before and constitutes a new family, nested among larger-bodied seaweed lineages. Endolithic clade #1 is related to *Pseudochlorodesmis*, which forms small turfs growing out of hard substrata, and it is known for having a problematic classification and high cryptic diversity^38^. Our results suggest that this lineage consists of primarily endolithic algae that only occasionally grow out of their rock but retain most of their biomass inside of it. Likewise, the two OTUs matching the macroalgae species *Halimeda discoidea* and *H. micronesica*, both of which were present in the areas where we collected, suggests *Halimeda* species have an endolithic life stage. Although the possibility of a contamination cannot be discarded entirely (e.g. the possibility of spores or gametes in the seawater), the high abundance of reads and their presence in several samples, even after rigorous quality control, indicate that this is unlikely. Endolithic “*Conchocelis*” stages have been described for red algal seaweeds^39^ but never for *Halimeda*. The life cycle of *Halimeda* has never been completed in culture^40^, perhaps because of unknown life stages such as these. *Halimeda* and many of the green algal endolithic lineages found here have also been sequenced from limestone substrates in a recently published study^37^.

We also retrieved OTUs related to three algae species that are known to bore into limestone: two OTUs are closely related to *Phaeophila dendroides*, two to *Ochlochaete hystrix* and two are related to *Ulvella* spp. *Phaeophila* is known to bore into coral skeletons, although to a lesser extent than *Ostreobium*^41^. *Ulvella* species have been reported as endophytic in other algae and as a pathogenic species in the skeleton of gorgonian corals^42^,^43^. *Ochlochaete hystrix* was found growing in shells^39^. This is the first time that *Ulvella* and *Ochlochaete* are reported in live stony coral skeletons.

When observing unexpected results such as the massive biodiversity of *Ostreobium* species and the presence of macroalgal species in an endolithic environment, it is desirable to address potential sequencing artifacts and contamination issues. Potential sources of contamination include the living coral tissue and the surrounding water. We have taken precautions to limit these sources of contamination in the field and during data processing. A good indication that our results do not result from spurious contaminations is that they are relatively abundant in multiple samples. Methodological artifacts include tag jumping and chimera formation^44^,^45^, and we have taken several precautions to avoid an overestimation of biodiversity due to these potential problems: 1) we apply a conservative similarity threshold to cluster OTUs; 2) we use a conservative de novo OTU clustering method (UPARSE) that is efficient in filtering chimeras without a reference database^46^; 3) our pipeline only keeps OTUs if they exceed 5 reads across the entire dataset; 4) our pipeline only keeps OTUs in individual samples if they exceed 2 reads in that sample; 5) we use a variety of controls including 10 mock extractions and 6 PCR negative controls. Due to these precautions, we may underestimate the endolithic microbial diversity but we rather err on the side of caution.

### Ecology and Evolution

Our phylogenetic analyses show that the *Ostreobium* clade originated in the Ordovician, around 500 my ago (Fig. 3 and^47^), which is in agreement with the oldest trace fossils attributed to this alga^48^. Although this pre-dates the existence of modern scleractinian corals, traces of ancestral *Ostreobium* lineages have been found in limestone rocks formed by extinct rugose corals, shells and stromatoporoids^48^,^49^, attesting the old origins of the endolithic lifestyle. Our results show that multiple *Ostreobium* lineages survived the Permian mass extinction and diversified after the Triassic, together with the rise of scleractinian reefs^50^. The appearance of endolithic clades #1 and #2 falls in the late Paleozoic, clade #1 diversified in the Mesozoic while clade #2 continued to diversify during the Cenozoic.

The ability to bore into coral skeletons evolved independently over 20 times in 12 Ulvophycean families (Fig. 3 and Supplementary Fig. S3 for *Cladophora*). This is surprising because *Ostreobium* and *Phaeophila* were thought to be the only green algae able to live within skeletons of live corals. The skeleton is an extreme environment for algae due to low light conditions and exposure to daily fluctuations of pH and oxygen levels caused by the holobiont’s photosynthesis and respiration^5^. The endolithic niche also varies depending on the coral species and external environmental conditions. It is therefore reasonable to expect that the endolithic lineages discovered here are not homogeneously distributed among these different niches. Indeed, the study of Gutner-Hoch and Fine^13^ suggests niche differentiation across depth gradients in the distribution of *Ostreobium* genotypes and some species-specific associations with corals.

The effects of diversity and distribution of endolithic algae on the coral holobiont is still to be investigated. For example, tolerance to thermal stress in some coral species is partly dependent on the relative abundance of certain *Symbiodinium* types, which can change in response to environmental perturbations^51^. Endolithic algal biomass within corals increases under elevated temperature and *p*CO_2_^8^, but it is unknown whether relative abundances of different lineages change, as is the case for *Symbiodinium*, and whether these different lineages have different ecological roles in the holobiont. By uncovering the diversity of endolithic algae, we set the stage and present methods to investigate these ecological interactions in detail. Scleractinian corals have been associated with endolithic algae since early in their evolution (Fig. 3), hence one would expect to find a variety of symbiotic associations ranging from mutualism to amensalism and perhaps parasitism, but these are yet to be discovered.

### Conclusion and perspectives

This study shows that metabarcoding surveys of coral-associated microbiomes based only on 16S rDNA or 18S rDNA underestimate the diversity of entire families of organisms, some of which have critical roles in the holobiont. We put forward the use of a cost effective multi-marker approach for more comprehensive biodiversity surveys. Our results reveal that both prokaryotic (e.g. cyanobacteria) and eukaryotic members of the microbiome within coral skeletons are more diverse than previously thought, offering interesting perspectives for future research on the interactions among these microorganisms.

By using a high-resolution marker and a phylogenetic framework we found six endolithic algal clades with divergence times close to the family level. Our results show that the oldest endolithic lineages originated *ca*. 500 million years ago, and the transition to a coral-endolithic lifestyle happened over 20 times in green algae evolution. With this baseline of their biodiversity and evolution at hand, it becomes possible to design ecophysiological experiments to investigate the adaptation of the different lineages to the endolithic niche. We are also applying the multi-marker environmental sequencing method in comparative ecology settings, for example, to study whether different coral species are associated with particular algal lineages and how coral-algal associations change as a function of ecological conditions. Besides helping to understand how these different lineages affect the holobiome resilience under environmental stress, this approach is likely to reveal a large number of species in other eukaryotic groups and assist to shed light on the darkest matter of microbial diversity.

## Materials and Methods

### Sampling and DNA isolation

The sampling was designed to set a solid baseline of the biodiversity and evolution of the endolithic community. We collected 132 coral skeleton samples from a wide variety of habitats and coral genera in Australia and Papua New Guinea (Supplementary Table 1). We chose not to focus our collections on systematic samples for comparative studies (i.e. multiple replicates for beta diversity analysis) at this stage. Instead, we targeted at a broad diversity of coral species and ecological conditions (depth, microhabitat) to increase chances of detecting different endolithic species.

After collection, the coral living tissue was removed and samples were stored in RNAlater or 100% ethanol. The environmental DNA was extracted using the Wizard Genomic DNA Purification Kit (Promega) or a phenol-chloroform protocol (Supplementary Materials). Although different DNA isolation protocols may be biased towards extracting certain groups of organisms more than others, we chose to analyse these samples together because we do not perform any comparative (e.g. beta-diversity) analysis that could be negatively affected by it. Ten mock DNA extractions were performed together with the samples DNA isolation to detect possible laboratory contaminants.

### Library preparation

We used a two-step PCR procedure to prepare Illumina sequencing libraries for multiple samples and markers. In order to add complexity to the library we added 0–3 random base pairs at the 5′ end of the primers^52^, followed by an overhang tail of 33 bp (Supplementary Table S2). We replaced a commonly used commercial kit (Nextera Index kit) by custom made oligos containing dual indices (8 bp) and Illumina adapters (Supplementary Table S2), which are ligated to the amplicons in the 2^nd^ PCR reaction. The details about the primers and both PCR amplification steps use here are given in the Supplementary Materials. Negative controls for all PCRs were sequenced and OTUs from those libraries were excluded in step 11 of the data processing workflow described below. We purified the samples using home-made magnetic beads as described in Rohland and Reich^27^, quantified, normalized and pooled the samples together (See Supplementary Materials for details). The libraries were sequenced with the Illumina MiSeq platform (2 × 300 bp paired end reads).

### Data processing pipeline

The steps used to process the multi-gene dataset, perform the quality control and OTU clustering were (see also Fig. 1B and Supplementary Materials for details):

1. Remove the reverse complement of adapters from short amplicons.
2. Separate genes into different files. With our library preparation design, the MiSeq run yields one file containing all four amplicons per sample, which can be teased apart based on primers sequence.
3. Trim 3′ ends of reads to improve consensus quality.
4. Merge forward and reverse reads using FLASH^53^.
5. Filter merged reads based on a quality threshold (average of 35 per merged sequence) using PRINSEQ^54^.
6. Trim primers from merged reads. Sequences that do not meet a minimum length threshold and/or do not have the primer sequence at the 3′ and 5′ ends are excluded in order to ensure global trimming.
7. Format the sequence headers to include sample name, run name and read number, then generate one file per gene containing all samples.
8. Cluster OTUs with the UPARSE pipeline^46^. Based on the divergence of the *tuf*A gene among Bryopsidales we used a similarity threshold of 98% for OTU clustering in this marker, which is a conservative threshold for species level. For the other markers we used the default threshold of 97% (see Supplementary Materials).
9. Alignment using PyNAST^55^ for 16S and 18S rDNA sequences and MAFFT^56^ for 23S rDNA and *tuf*A.
10. Assign taxonomy using the Naïve Bayesian Classifier (RDP) implemented in QIIME^19^,^28^. We used RDP taxonomic assignments to: i) infer the abundance of reads assigned to the main microbial groups; and ii) pre-filter OTUs to build a green algae phylogeny: OTUs that were not classified as “Eukaryotic” were excluded from the *tufA* phylogenetic analysis. Likewise, only OTUs classified as “Chloroplast” in the 16S rDNA dataset were included in the 16S phylogeny.
11. Filter OTUs found in negative controls.
12. Filter OTU table by minimum count (2) of reads per OTU per sample.
13. Filter rare OTUs and produce final filtered OTU fasta file.
14. Produce final OTU table and statistics.

### Phylogenetic analysis

In order to place the OTUs in a green algal phylogeny, we created reference alignments containing *tuf*A, *rbc*L, 18S, 16S and 23S rDNA sequences of green algae (Supplementary Materials). We subsequently added the sequence data for the OTUs to the multi-locus alignment, producing one alignment per amplicon (4833–9489 bp). We aligned the OTUs with the reference sequences using Geneious^57^ and MAFFT^56^. We used Partitioned Model Tester v.1.03 (https://github.com/hverbruggen/PMT) to identify the best-fit model of molecular evolution and partitioning strategy, then reconstructed the phylogeny using RAxML^58^, with *Prasinococcus capsulatus* as outgroup. OTUs that did not fall within the core Chlorophyta were excluded from the analysis.

We calibrated the phylogeny containing the *tuf*A OTUs in geological time with the PhyloBayes program^59^ using node ages estimated in a previous study^47^. To infer the origins of the coral skeleton-boring nature, we classified taxa into coral-endolithic or non-coral-endolithic. Besides the OTUs retrieved here, the following species were classified as coral-endolithic: *Phaeophila dendroides, Ulvella endozoica* and *Ostreobium spp.*, which have been reported from coral skeletons^4^,^41^,^42^ and *Halimeda discoidea* and *H. micronesica*, which have identical *tuf*A sequences to two retrieved OTUs. We estimated the ancestral states with 1000 simulations of stochastic mapping using the R package phytools^60^, and plotted the average log-likelihood of the ancestral states along the tree with TreeGradients v.1.03 (available at www.phycoweb.net).

## Additional Information

**How to cite this article**: Rossetto Marcelino, V. and Verbruggen, H. Multi-marker metabarcoding of coral skeletons reveals a rich microbiome and diverse evolutionary origins of endolithic algae. *Sci. Rep*. **6**, 31508; doi: 10.1038/srep31508 (2016).

## Supplementary Material

Supplementary Information

## References

[b1] Rosenberg E., Koren O., Reshef L., Efrony R. & Zilber-Rosenberg I. The role of microorganisms in coral health, disease and evolution. Nat. Rev. Microbiol. 5, 355–362 (2007). 17384666 10.1038/nrmicro1635

[b2] Blackall L. L., Wilson B. & van Oppen M. J. H. Coral-the world’s most diverse symbiotic ecosystem. Mol. Ecol. 24, 5330–5347 (2015). 26414414 10.1111/mec.13400

[b3] Verbruggen H. & Tribollet A. Boring algae. Curr. Biol. 21, R876–R877 (2011). 22075423 10.1016/j.cub.2011.09.014

[b4] Tribollet A. The boring microflora in modern coral reef ecosystems: a review of its roles In Curr. Dev. Bioerosion (eds Wisshak M. & Tapanila L.) 67–94 (Springer Berlin Heidelberg, 2008).

[b5] Shashar N. & Stambler N. Endolithic algae within corals - life in an extreme environment. J. Exp. Mar. Bio. Ecol. 163, 277–286 (1992).

[b6] Tribollet A. Dissolution of dead corals by euendolithic microorganisms across the northern Great Barrier Reef (Australia). Microb. Ecol. 55, 569–80 (2008). 17690835 10.1007/s00248-007-9302-6

[b7] Grange J. S., Rybarczyk H. & Tribollet A. The three steps of the carbonate biogenic dissolution process by microborers in coral reefs (New Caledonia). Environ. Sci. Pollut. Res. 22, 13625–13637 (2015). 10.1007/s11356-014-4069-z 25592911

[b8] Reyes-Nivia C., Diaz-Pulido G., Kline D., Hoegh-Guldberg O. & Dove S. Ocean acidification and warming scenarios increase microbioerosion of coral skeletons. Glob. Chang. Biol. 19, 1919–1929 (2013). 23505093 10.1111/gcb.12158

[b9] Yamazaki S. S., Nakamura T. & Yamasaki H. Photoprotective role of endolithic algae colonized in coral skeleton for the host photosynthesis In Photosynth. Energy from Sun (eds Allen J., Gantt E., Golbeck J. H. & Osmond B.) 1391–1395 (Springer Netherlands, 2008).

[b10] Fine M. & Loya Y. Endolithic algae: an alternative source of photoassimilates during coral bleaching. Proc. R. Soc. B-Biological Sci. 269, 1205–10 (2002). 10.1098/rspb.2002.1983 PMC1691023 12065035

[b11] Vroom P. & Smith C. The challenge of siphonous green algae. Am. Sci. 89, 524 (2001).

[b12] Verbruggen H. Morphological complexity, plasticity, and species diagnosability in the application of old species names in DNA-based taxonomies. J. Phycol. 50, 26–31 (2014). 26988006 10.1111/jpy.12155

[b13] Gutner-Hoch E. & Fine M. Genotypic diversity and distribution of *Ostreobium quekettii* within scleractinian corals. Coral Reefs 30, 643–650 (2011).

[b14] Taberlet P., Coissac E., Pompanon F., Brochmann C. & Willerslev E. Towards next-generation biodiversity assessment using DNA metabarcoding. Mol. Ecol. 21, 2045–50 (2012). 22486824 10.1111/j.1365-294X.2012.05470.x

[b15] Rappé M. S. & Giovannoni S. J. The uncultured microbial majority. Annu. Rev. Microbiol. 57, 369–394 (2003). 14527284 10.1146/annurev.micro.57.030502.090759

[b16] Marcy Y. . Dissecting biological ‘dark matter’ with single-cell genetic analysis of rare and uncultivated TM7 microbes from the human mouth. Proc. Natl. Acad. Sci. 104, 11889–11894 (2007). 17620602 10.1073/pnas.0704662104 PMC1924555

[b17] Lema K. A., Willis B. L. & Bourne D. G. Amplicon pyrosequencing reveals spatial and temporal consistency in diazotroph assemblages of the *Acropora millepora* microbiome. Environ. Microbiol. 16, 3345–3359 (2014). 24373029 10.1111/1462-2920.12366

[b18] Kirkham A. R. . Basin-scale distribution patterns of photosynthetic picoeukaryotes along an Atlantic Meridional Transect. Environ. Microbiol. 13, 975–990 (2011). 21219562 10.1111/j.1462-2920.2010.02403.x

[b19] Wang Q., Garrity G. M., Tiedje J. M. & Cole J. R. Naive Bayesian Classifier for rapid assignment of rRNA sequences into the new bacterial taxonomy. Appl. Environ. Microbiol. 73, 5261–5267 (2007). 17586664 10.1128/AEM.00062-07 PMC1950982

[b20] Altschul S. F., Gish W., Miller W., Myers E. W. & Lipman D. J. Basic local alignment search tool. J. Mol. Biol. 215, 403–410 (1990). 2231712 10.1016/S0022-2836(05)80360-2

[b21] Yilmaz P. & Glöckner F. O. Metagenomes: 23S Sequences In Encycl. Metagenomics: Genes, Genomes and Metagenomes: Basics, Methods, Databases and Tools (ed. Nelson K. E.) 396–402 (Springer US, 2015)

[b22] Klindworth A. . Evaluation of general 16S ribosomal RNA gene PCR primers for classical and next-generation sequencing-based diversity studies. Nucleic Acids Res. 41, e1 (2013). 22933715 10.1093/nar/gks808 PMC3592464

[b23] Porazinska D. L. . Evaluating high-throughput sequencing as a method for metagenomic analysis of nematode diversity. Mol. Ecol. Resour. 9, 1439–1450 (2009). 21564930 10.1111/j.1755-0998.2009.02611.x

[b24] Presting G. G. Identification of conserved regions in the plastid genome: implications for DNA barcoding and biological function. Can. J. Bot. 84, 1434–1443 (2006).

[b25] Saunders G. & Kucera H. An evaluation of *rbc*L. *tuf*A. UPA. LSU and ITS as DNA barcode markers for the marine green macroalgae. Cryptogam. Algol. 31, 487–528 (2010).

[b26] Gilbert J. A., Jansson J. K. & Knight R. The Earth Microbiome project: successes and aspirations. BMC Biol. 12, 69 (2014). 25184604 10.1186/s12915-014-0069-1 PMC4141107

[b27] Rohland N. & Reich D. Cost-effective, high-throughput DNA sequencing libraries for multiplexed target capture. Genome Res. 22, 939–46 (2012). 22267522 10.1101/gr.128124.111 PMC3337438

[b28] Caporaso J. G. . QIIME allows analysis of high-throughput community sequencing data. Nat. Methods 7, 335–336 (2010). 20383131 10.1038/nmeth.f.303 PMC3156573

[b29] Ainsworth T. . The coral core microbiome identifies rare bacterial taxa as ubiquitous endosymbionts. ISME J. 9, 2261–2274 (2015). 10.1038/ismej.2015.39 PMC4579478 25885563

[b30] Li J. . Bacterial dynamics within the mucus, tissue and skeleton of the coral *Porites lutea* during different seasons. Sci. Rep. 4, 7320 (2014). 25475855 10.1038/srep07320 PMC4256709

[b31] Andrews J. H. The pathology of marine algae. Biol. Rev. 51, 211–252 (1976).

[b32] Kittelmann S. . Simultaneous amplicon sequencing to explore co-occurrence patterns of bacterial, archaeal and eukaryotic microorganisms in rumen microbial communities. Plos One 8, e47879 (2013). 23408926 10.1371/journal.pone.0047879 PMC3568148

[b33] La Claire J. W. & Wang J. S. Structural characterization of the terminal domains of linear plasmid-like DNA from the green alga *Ernodesmis* (Chlorophyta). J. Phycol. 40, 1089–1097 (2004).

[b34] Fernando S. C. . Microbiota of the major south atlantic reef building coral *Mussismilia*. Microb. Ecol. 69, 267–280 (2014). 25213651 10.1007/s00248-014-0474-6

[b35] Yang S.-H. . Prevalence of potential nitrogen-fixing, green sulfur bacteria in the skeleton of reef-building coral *Isopora palifera*. Limnol. Oceanogr. 61, 1078–1086 (2016).

[b36] Behrendt L. . Endolithic chlorophyll d-containing phototrophs. ISME J. 5, 1072–1076 (2011). 21160540 10.1038/ismej.2010.195 PMC3131860

[b37] Sauvage T., Schmidt W. E., Suda S. & Fredericq S. A metabarcoding framework for facilitated survey of endolithic phototrophs with *tuf*A. BMC Ecol. 16, 8 (2016). 26965054 10.1186/s12898-016-0068-x PMC4785743

[b38] Verbruggen H. . Phylogenetic analysis of *Pseudochlorodesmis* strains reveals cryptic diversity above the family level in the siphonous green algae (Bryopsidales, Chlorophyta). J. Phycol. 45, 726–731 (2009). 27034048 10.1111/j.1529-8817.2009.00690.x

[b39] Nielsen R. Marine algae within calcareous shells from New Zealand. New Zeal. J. Bot. 25, 425–438 (1987).

[b40] Meinesz A. Sur le cycle de l’*Halimeda tune* (Ellis et Solander) Lamouroux (Udoteacee, Caulerpale). Compte Rendu Hebd. des Séances l’Académie des Sci. Paris 275, 1363–1365 (1972).

[b41] Titlyanov E. A., Kiyashko S. I., Titlyanova T. V., Kalita T. L. & Raven J. A. δ13C and δ15N values in reef corals *Porites lutea* and *P. cylindrica* and in their epilithic and endolithic algae. Mar. Biol. 155, 353–361 (2008).

[b42] Goldberg W. M., Makemson J. C. & Colley S. B. *Entocladia endozoica* sp. nov., A pathogenic chlorophyte: structure, life history, physiology, and effect on its coral host. Biol. Bull. 166, 368 (1984).

[b43] Correa J. A. & McLachlan J. L. Endophytic algae of *Chondrus crispus* (Rhodophyta). V. Fine structure of the infection by *Acrochaete operculata* (Chlorophyta). Eur. J. Phycol. 29, 33–47 (1994).

[b44] Acinas S. G., Sarma-Rupavtarm R., Klepac-Ceraj V. & Polz M. F. PCR-Induced sequence artifacts and bias: insights from comparison of two 16s rRNA clone libraries constructed from the same sample. Appl. Environ. Microbiol. 71, 8966–8969 (2005). 16332901 10.1128/AEM.71.12.8966-8969.2005 PMC1317340

[b45] Schnell I. B., Bohmann K. & Gilbert M. T. P. Tag jumps illuminated - reducing sequence-to-sample misidentifications in metabarcoding studies. Mol. Ecol. Resour. 15, 1289–1303 (2015). 25740652 10.1111/1755-0998.12402

[b46] Edgar R. C. UPARSE: highly accurate OTU sequences from microbial amplicon reads. Nat. Methods 647, 1–5 (2013). 10.1038/nmeth.2604 23955772

[b47] Verbruggen H. . A multi-locus time-calibrated phylogeny of the siphonous green algae. Mol. Phylogenet. Evol. 50, 642–653 (2009). 19141323 10.1016/j.ympev.2008.12.018

[b48] Vogel K. & Brett C. E. Record of microendoliths in different facies of the Upper Ordovician in the Cincinnati Arch region USA: The early history of light-related microendolithic zonation. Palaeogeogr. Palaeoclimatol. Palaeoecol. 281, 1–24 (2009).

[b49] Vogel K. Bioeroders in fossil reefs. Facies 28, 109–113 (1993).

[b50] Stanley G. D. The evolution of modern corals and their early history. Earth-Science Rev. 60, 195–225 (2003).

[b51] Berkelmans R. & van Oppen M. J. H. The role of zooxanthellae in the thermal tolerance of corals: a ‘nugget of hope’ for coral reefs in an era of climate change. Proc. R. Soc. B Biol. Sci. 273, 2305–2312 (2006). 10.1098/rspb.2006.3567 PMC1636081 16928632

[b52] Tremblay J. . Primer and platform effects on 16S rRNA tag sequencing. Front. Microbiol. 6, 1–15 (2015). 26300854 10.3389/fmicb.2015.00771 PMC4523815

[b53] Magoč T. & Salzberg S. L. FLASH: Fast length adjustment of short reads to improve genome assemblies. Bioinformatics 27, 2957–2963 (2011). 21903629 10.1093/bioinformatics/btr507 PMC3198573

[b54] Schmieder R. & Edwards R. Quality control and preprocessing of metagenomic datasets. Bioinformatics 27, 863–864 (2011). 21278185 10.1093/bioinformatics/btr026 PMC3051327

[b55] Caporaso J. G. . PyNAST: a flexible tool for aligning sequences to a template alignment. Bioinformatics 26, 266–267 (2010). 19914921 10.1093/bioinformatics/btp636 PMC2804299

[b56] Katoh K., Misawa K., Kuma K. & Miyata T. MAFFT: a novel method for rapid multiple sequence alignment based on fast Fourier transform. Nucleic Acids Res. 30, 3059–3066 (2002). 12136088 10.1093/nar/gkf436 PMC135756

[b57] Kearse M. . Geneious Basic: An integrated and extendable desktop software platform for the organization and analysis of sequence data. Bioinformatics 28, 1647–1649 (2012). 22543367 10.1093/bioinformatics/bts199 PMC3371832

[b58] Stamatakis A. RAxML-VI-HPC: maximum likelihood-based phylogenetic analyses with thousands of taxa and mixed models. Bioinformatics 22, 2688–2690 (2006). 16928733 10.1093/bioinformatics/btl446

[b59] Lartillot N., Lepage T. & Blanquart S. PhyloBayes 3: a Bayesian software package for phylogenetic reconstruction and molecular dating. Bioinformatics 25, 2286–2288 (2009). 19535536 10.1093/bioinformatics/btp368

[b60] Revell L. J. phytools: an R package for phylogenetic comparative biology (and other things). Methods Ecol. Evol. 3, 217–223 (2012).

