# Supplementary material for: Multi-marker metabarcoding of coral skeletons reveals a rich microbiome and diverse evolutionary origins of endolithic algae
Source: Sci Rep. 2016 Aug 22;6:31508. doi: 10.1038/srep31508 (PMC4992875; doi:10.1038/srep31508)
Supplement: Supplementary Information [file srep31508-s1.pdf]

Supplementary Materials

Multi-marker metabarcoding of coral skeletons reveals a rich microbiome and diverse evolutionary origins of endolithic algae.

Vanessa Rossetto Marcelino and Heroen Verbruggen

Sampling and DNA isolation

Supplementary Table S1: Coral skeleton samples and locality.

Sample	Country	Locality	Coral host
**HV04438.R3**	Papua New Guinea	Kavieng	Unidentified stony coral
**HV04837.R3**	Papua New Guinea	Kavieng	*Favites sp.*
**HV04841.R3**	Papua New Guinea	Kavieng	*Porites sp.*
**HV04853.R3**	Papua New Guinea	Kavieng	*Millepora sp.*
**HV04854.R3**	Papua New Guinea	Kavieng	*Goniastrale pectinata*
**HV04857.R3**	Papua New Guinea	Kavieng	*Porites sp.*
**HV05003.R3**	Papua New Guinea	Kavieng	*Goniastrea sp.*
**HV05004b.R3**	Papua New Guinea	Kavieng	*Platygyra sp.*
**HV05004c.R3**	Papua New Guinea	Kavieng	*Platygyra sp.*
**HV05005.R3**	Papua New Guinea	Kavieng	*Porites sp.*
**HV05006a.R3**	Papua New Guinea	Kavieng	*Goniastrea edwardsi*
**HV05006b.R3**	Papua New Guinea	Kavieng	*Goniastrea edwardsi*
**HV05007.R3**	Papua New Guinea	Kavieng	*Diploastrea heliopora*
**HV05008.R3**	Papua New Guinea	Kavieng	*Leptoseris sp.*
**HV05009.R3**	Papua New Guinea	Kavieng	*Favites russelli*
**HV05010.R3**	Papua New Guinea	Kavieng	*Madracis sp.*
**HV05011.R3**	Papua New Guinea	Kavieng	*Oxypora lacera*
**HV05012.R3**	Papua New Guinea	Kavieng	*Leptoseris striatus*
**HV05013.R3**	Papua New Guinea	Kavieng	*Pectinia sp.*
**HV05014.R3**	Papua New Guinea	Kavieng	*Leptoseris striata*
**HV05015.R3**	Papua New Guinea	Kavieng	*Porites profundus*
**HV05016.R3**	Papua New Guinea	Kavieng	*Goniastrea edwardsii*
**HV05017.R3**	Papua New Guinea	Kavieng	*Goniastrea edwardsii*
**HV05019.R3**	Papua New Guinea	Kavieng	*Favites halicora*
**HV05020.R3**	Papua New Guinea	Kavieng	*Pachyseris sp.*
**HV05021.R3**	Papua New Guinea	Kavieng	*Goniastrea edwardsii*
**HV05022.R3**	Papua New Guinea	Kavieng	*Platygyra lamellina*
**HV05023.R3**	Papua New Guinea	Kavieng	*Porites australiensis*
**HV05024.R3**	Papua New Guinea	Kavieng	*Lobophyllia sp.*
**HV05025.R3**	Papua New Guinea	Kavieng	*Symphyllia valenciennesi*
**HV05026.R3**	Papua New Guinea	Kavieng	*Goniastrea edwardsii*
**HV05028.R3**	Papua New Guinea	Kavieng	*Stylophora pistillata*
**HV05029.R3**	Papua New Guinea	Kavieng	*Porites australiensis*
**HV05031.R3**	Papua New Guinea	Kavieng	*Pachyseris sp.*
**HV05032.R3**	Papua New Guinea	Kavieng	*Pertinia sp.*
**HV05034.R3**	Papua New Guinea	Kavieng	*Porites sp.*
**HV05035.R3**	Papua New Guinea	Kavieng	*Echinopora hirsutissima*
**HV05037.R3**	Papua New Guinea	Kavieng	*Merulina ampliata*
**HV05038.R3**	Papua New Guinea	Kavieng	*Pachyseris sp.*
**HV05039.R3**	Papua New Guinea	Kavieng	*Merulina ampliata*
**HV05040c.R3**	Papua New Guinea	Kavieng	*Porites sp.*
**HV05041a.R3**	Papua New Guinea	Kavieng	*Galaxea astreata*
**HV05041b.R3**	Papua New Guinea	Kavieng	*Galaxea astreata*
**HV05042.R3**	Papua New Guinea	Kavieng	*Diploastrea heliopora*
**HV05043.R3**	Papua New Guinea	Kavieng	*Echinopora hirsutissima*

**HV05044.R3**	Papua New Guinea	Kavieng	*Lobophyllia sp.*
**HV05045.R3**	Papua New Guinea	Kavieng	*Oxypora lacera*
**HV05046.R3**	Papua New Guinea	Kavieng	*Leptoseris troglodyta*
**HV05047.R3**	Papua New Guinea	Kavieng	*Leptoseris mycetoseroides*
**PHV207.R1**	Papua New Guinea	PNG	Unidentified stony coral
**PHV237.R1**	Papua New Guinea	PNG	Unidentified stony coral
**PHV570.R1**	Papua New Guinea	PNG	Unidentified stony coral
**PHV882.R1**	Papua New Guinea	PNG	Unidentified stony coral
**VRM0028.R1**	Australia	Western Australia	*Montipora sp.*
**VRM0032.R1**	Australia	Western Australia	*Montipora sp.*
**VRM0036.R1**	Australia	Western Australia	Unidentified stony coral
**VRM0039.R2**	Australia	Western Australia	*Cyphastrea sp.*
**VRM0040.R2**	Australia	Western Australia	*Goniastrea sp.*
**VRM0042.R2**	Australia	Western Australia	*Goniastrea sp.*
**VRM0043.R2**	Australia	Western Australia	*Leptoria sp.*
**VRM0044.R2**	Australia	Western Australia	Unidentified stony coral
**VRM0045.R2**	Australia	Western Australia	*Montastrea sp.*
**VRM0046.R2**	Australia	Western Australia	Unidentified stony coral
**VRM0048.R2**	Australia	Western Australia	*Goniastrea sp.*
**VRM0051.R2**	Australia	Western Australia	*Porites sp.*
**VRM0052.R2**	Australia	Western Australia	*Porites sp.*
**VRM0053.R2**	Australia	Western Australia	*Porites sp.*
**VRM0054.R2**	Australia	Western Australia	*Porites sp.*
**VRM0055.R2**	Australia	Western Australia	*Porites sp.*
**VRM0056.R2**	Australia	Western Australia	*Porites sp.*
**VRM0057.R2**	Australia	Western Australia	*Porites sp.*
**VRM0058.R2**	Australia	Western Australia	*Porites sp.*
**VRM0059.R2**	Australia	Western Australia	*Porites sp.*
**VRM0060.R1**	Australia	Western Australia	*Porites sp.*
**VRM0061.R2**	Australia	Western Australia	*Porites sp.*
**VRM0062.R2**	Australia	Western Australia	*Porites sp.*
**VRM0063.R2**	Australia	Western Australia	*Porites sp.*
**VRM0064.R2**	Australia	Western Australia	*Porites sp.*
**VRM0065.R2**	Australia	Western Australia	*Porites sp.*
**VRM0066.R1**	Australia	Western Australia	*Porites sp.*
**VRM0067.R1**	Australia	Western Australia	*Porites sp.*
**VRM0068.R2**	Australia	Western Australia	*Porites sp.*
**VRM0069.R2**	Australia	Western Australia	*Porites sp.*
**VRM0070.R2**	Australia	Western Australia	*Porites sp.*
**VRM0071.R2**	Australia	Western Australia	*Porites sp.*
**VRM0072.R2**	Australia	Western Australia	*Porites sp.*
**VRM0073.R2**	Australia	Western Australia	*Porites sp.*
**VRM0074.R2**	Australia	Western Australia	*Porites sp.*
**VRM0076.R2**	Australia	Western Australia	*Porites sp.*
**VRM0077.R2**	Australia	Western Australia	*Porites sp.*
**VRM0078.R2**	Australia	Western Australia	*Porites sp.*
**VRM0079.R2**	Australia	Western Australia	*Porites sp.*
**VRM0080.R2**	Australia	Western Australia	*Porites sp.*
**VRM0081.R1**	Australia	Western Australia	*Porites sp.*
**VRM0082.R2**	Australia	Western Australia	*Porites sp.*
**VRM0083.R2**	Australia	Western Australia	*Porites sp.*
**VRM0084.R2**	Australia	Western Australia	*Porites sp.*
**VRM0085.R2**	Australia	Western Australia	*Porites sp.*
**VRM0086.R1**	Australia	Western Australia	*Porites sp.*
**VRM0087.R1**	Australia	Western Australia	*Porites sp.*
**VRM0090.R1**	Australia	Western Australia	*Porites sp.*
**VRM0091.R1**	Australia	Western Australia	*Montipora sp.*
**VRM0096.R1**	Australia	Western Australia	*Porites sp.*
**VRM0097.R2**	Australia	Western Australia	*Porites sp.*
**VRM0098.R1**	Australia	Western Australia	*Porites sp.*
**VRM0099.R1**	Australia	Western Australia	*Porites sp.*

**VRM0100.R1**	Australia	Western Australia	*Porites sp.*
**VRM0101.R2**	Australia	Western Australia	*Porites sp.*
**VRM0102.R2**	Australia	Western Australia	*Porites sp.*
**VRM0103.R2**	Australia	Western Australia	*Porites sp.*
**VRM0104.R2**	Australia	Western Australia	*Porites sp.*
**VRM0105.R2**	Australia	Western Australia	*Porites sp.*
**VRM0106.R1**	Australia	Western Australia	*Porites sp.*
**VRM0107.R2**	Australia	Western Australia	*Porites sp.*
**VRM0108.R2**	Australia	Western Australia	*Porites sp.*
**VRM0109.R2**	Australia	Western Australia	*Porites sp.*
**VRM0110.R2**	Australia	Western Australia	*Porites sp.*
**VRM0111.R2**	Australia	Western Australia	*Porites sp.*
**VRM0112.R2**	Australia	Western Australia	*Porites sp.*
**VRM0113.R2**	Australia	Western Australia	*Porites sp.*
**VRM0114.R2**	Australia	Western Australia	*Porites sp.*
**VRM0115.R1**	Australia	Western Australia	*Porites sp.*
**VRM0116.R2**	Australia	Western Australia	*Porites sp.*
**VRM0117.R2**	Australia	Western Australia	*Porites sp.*
**VRM0118.R2**	Australia	Western Australia	*Porites sp.*
**VRM0119.R2**	Australia	Western Australia	*Porites sp.*
**VRM0120.R1**	Australia	Western Australia	*Porites sp.*
**VRM0121.R1**	Australia	Western Australia	*Porites sp.*
**VRM0122.R1**	Australia	Western Australia	*Porites sp.*
**VRM0123.R1**	Australia	Western Australia	*Porites sp.*
**VRM0124.R1**	Australia	Western Australia	*Porites sp.*
**VRM0190.R1**	Australia	Queensland	*Pocillopora sp.*

After collection, the coral tissue was removed with pliers and razor blades and samples were stored in RNAlater or 100% ethanol. The DNA was extracted using either: (1) a modified phenol-chloroform protocol — see Cremen *et al.* (2016); our only modification was to use phenol/chloroform/isoamyl alcohol [25:24:1] (instead of chloroform/isoamyl alcohol only) in the first extraction step. Or (2) the Wizard Genomic DNA Purification Kit (Promega). More amplification success and DNA yield was obtained using the kit with a small modification: incubating a piece of coral skeleton (ca. 80mm^3) in the lysis buffer for 3 hours without grinding the sample, then proceeding with the manufacturer's instructions for plant tissue.

Library preparation:

The amplicons and respective primers used here were:

16S rDNA: We used either the 515f/806r (Caporaso *et al.*, 2012; Gilbert *et al.*, 2014) or the S-D-Bact-0341-b-S-17/S-D-Bact-0785-a-A-21 (Klindworth *et al.*, 2013) primer pairs to PCR-amplify this marker. The amplicons generated by these 2 primer pairs overlap in the V3-V4 region, so we used only this overlapping region — the sequence length after trimming primer sequences was, on average, 225 base pairs.

18S rDNA: We used the NF1/18 Sr2b primer combination (Porazinska *et al.*, 2009).

23S rDNA: We used the algal specific primer pair p23SrV_r1/p23SrV_f1, which PCR-amplifies the Universal Plastid Amplicon (Presting 2006).

***tuf*A:** We used primers *tuf*AR (Fama *et al.*, 2002) and a forward primer designed here for *Ostreobium* (Oq-tuf: ACN GGN CGN GGN ACN GT), which has several ambiguous bases in order to amplify a larger range of green algae species.

Supplementary Table S2: 1st PCR primers design†

	Tail 1	Ns (0-3bp)	Primer (e.g. 16S 515/806)
**Forward**	TCGTCGGCAGCGTCAGATGTGTATAAGAGACAG	NNN	[GTGCCAGCMGCCGCGGTAA]
**Reverse**	GTCTCGTGGGCTCGGAGATGTGTATAAGAGACAG	NNN	[GGACTACHVGGGTWTCTAAT]

† Oligonucleotide sequences © 2007-2012 Illumina, Inc. All rights reserved - Derivative works created by Illumina customers are authorized for use with Illumina instruments and products only. All other uses are strictly prohibited.

Supplementary Table S3: 2nd PCR oligonucleotides†.

Forward Illumina adapter	F Indices	Tail_2
A*ATGATACGGCGACCACCGAGATCTACAC	TAGATCGC	TCGTCGGCAGCGTC
A*ATGATACGGCGACCACCGAGATCTACAC	CTCTCTAT	TCGTCGGCAGCGTC
A*ATGATACGGCGACCACCGAGATCTACAC	TATCCTCT	TCGTCGGCAGCGTC
A*ATGATACGGCGACCACCGAGATCTACAC	AGAGTAGA	TCGTCGGCAGCGTC
A*ATGATACGGCGACCACCGAGATCTACAC	GTAAGGAG	TCGTCGGCAGCGTC
A*ATGATACGGCGACCACCGAGATCTACAC	ACTGCATA	TCGTCGGCAGCGTC
A*ATGATACGGCGACCACCGAGATCTACAC	AAGGAGTA	TCGTCGGCAGCGTC
A*ATGATACGGCGACCACCGAGATCTACAC	CTAAGCCT	TCGTCGGCAGCGTC
**Reverse Illumina adapter**	**R Indices**	**Tail_2**
C*AAGCAGAAGACGGCATACGAGAT	TAAGGCGA	GTCTCGTGGGCTCGG
C*AAGCAGAAGACGGCATACGAGAT	CGTACTAG	GTCTCGTGGGCTCGG
C*AAGCAGAAGACGGCATACGAGAT	AGGCAGAA	GTCTCGTGGGCTCGG
C*AAGCAGAAGACGGCATACGAGAT	TCCTGAGC	GTCTCGTGGGCTCGG
C*AAGCAGAAGACGGCATACGAGAT	GGACTCCT	GTCTCGTGGGCTCGG
C*AAGCAGAAGACGGCATACGAGAT	TAGGCATG	GTCTCGTGGGCTCGG
C*AAGCAGAAGACGGCATACGAGAT	CTCTCTAC	GTCTCGTGGGCTCGG
C*AAGCAGAAGACGGCATACGAGAT	CAGAGAGG	GTCTCGTGGGCTCGG
C*AAGCAGAAGACGGCATACGAGAT	GCTACGCT	GTCTCGTGGGCTCGG
C*AAGCAGAAGACGGCATACGAGAT	CGAGGCTG	GTCTCGTGGGCTCGG
C*AAGCAGAAGACGGCATACGAGAT	AAGAGGCA	GTCTCGTGGGCTCGG
C*AAGCAGAAGACGGCATACGAGAT	GTAGAGGA	GTCTCGTGGGCTCGG

*Indicates a phosphorothioate modification. † Oligonucleotide sequences © 2007-2012 Illumina, Inc. All rights reserved - Derivative works created by Illumina customers are authorized for use with Illumina instruments and products only. All other uses are strictly prohibited.

We amplified the four markers in separate reactions containing 0.2 mM dNTP mix, 0.5 µM forward and reverse primers, 2 mM $MgCl_2$, 0.4 µg/µl Bovine Serum Albumin, 1× PCR buffer and 0.25U of Platinum *Taq* DNA Polymerase (Invitrogen). The first PCR round consisted of: initial denaturation step at 94°C for 5 min, followed by 25 cycles of denaturation (94°C for 30 s), annealing (45 s) and extension (72°C for 30 s) and a final extension step at 72°C for 5 min for the ribosomal DNA markers. Annealing temperature was set at 50°C for primer pair 515f/806r, 55°C for p23SrV_r1/p23SrV_f1 and S-D-Bact-0341-b-S-17/S-D-Bact-0785-a-A-21 and 60°C for NF1/18Sr2b. Because *tuf*A is a coding gene, it has higher mutation rates (especially at 3rd codon positions) when compared to ribosomal DNA, therefore a touchdown step and a lower

annealing temperature is required (55—48°C for 14 cycles followed by 24 cycles at 48°C). Unspecific amplification does occur, but those are excluded in the analysis pipeline (e.g. steps 5, 6 and 9 of the pipeline).

For the second PCR we used the following conditions: initial denaturation step at 94°C for 5 min, followed by 8 cycles of denaturation (94°C at 30 s), annealing (55°C at 30 s) and extension (72°C at 30 s) and a final extension step at 72°C for 5 min. We purified the samples using home-made magnetic beads as described in Rohland and Reich (2012) and quantified the libraries using the Qubit fluorometer (Invitrogen). We produced libraries for three runs: the first run containing 48 samples (of which 5 were PCR controls or mock extractions), the second and third runs contained 96 samples (including 7 and 8 controls, respectively). The libraries were sequenced with the Illumina MiSeq platform (V3 kit - 2×300 bp PE reads) at the Centre for Translational Pathology, University of Melbourne. The runs generated sequences for all samples that had a successful PCR amplification (*ca.*1 uM or more). Not all samples successfully sequenced in these three runs are included in this study: here we included the 132 samples listed above, the remaining samples are part of a different study and will be published separately.

Costs calculation:

20 indexed oligos (Supplementary Table 3), with phosphorothioate modification, produced at 200 nmole scale by Bioneer Pacific = 425AUD (Including GST). Each 200 nmole oligonucleotide is sufficient for 800 reactions. Prices for plate orders.

Nextera kit with 96 Indices, 384 samples (FC-121-1012) = 1132 AUD.

Prices are from 2014.

1 Australian Dollar (AUD) ≈ 0.72 US Dollar (October 2015).

Data processing pipeline:

1. Remove the reverse complement of adapters from short amplicons. When the length of the reads is longer than the amplicon, you will get the reverse complement of the adapter sequenced in the 3' end of the read, which can influence the merging of the paired end sequences.
2. Separate genes into different files. With our library preparation design, the MiSeq run yields one file per sample, each containing all amplicons. The different amplicons are teased apart based on primers sequences in this step.
3. Trim 3' ends of reads (5 bases in forward reads and 20 bases in reverse reads) to improve consensus quality.

4. Merge forward and reverse reads using FLASH (Magoč and Salzberg, 2011).

5. Quality control: filter merged reads based on a quality threshold (average of 35 per merged read) using PRINSEQ (Schmieder and Edwards, 2011).

6. Trim primers from merged reads. Sequences that do not meet a minimum length threshold and/or do not have the exact primer sequence at the 3' and 5' ends are excluded from analysis in order to ensure quality (i.e., the sequence belong to the target gene) and global trimming (i.e., they start and end at the same position).

7. Format reads' identification and generate one file per gene containing all samples.

8. Run UPARSE pipeline (Edgar, 2013): dereplication, sort by size, cluster OTUs and produce OTU map. We chose UPARSE because other available software (e.g. Qiime and Mothur) seem to significantly overestimate the number of OTUs (Edgar, 2013). Based on the divergence of *tuf*A among Bryopsidales we used a similarity threshold of 98% for OTU clustering in this marker, which is a conservative threshold for species level (i.e., most Bryopsidales species are more similar than that, so at 98% the OTUs will be somewhere between species and genus level). We choose the 97% threshold for the other markers for two main reasons: 1) there is not enough information in literature about the rDNA markers similarity among Bryopsidales species, on the contrary, it is known that they do not have phylogenetic signal to distinguish them; 2) our aim was to compare how the normally used markers (with their commonly used thresholds) perform in distinguishing algae species.

9. Alignment: we used PyNAST (Caporaso *et al.*, 2010a) to align the 16S and 18S rDNA sequences. This aligner requires a reference database with aligned sequences and lots of gaps in the alignment. Due to the lack of such reference databases for 23S rDNA and *tuf*A, we chose MAFFT (Katoh *et al.*, 2002) to align 23S rDNA and *tuf*A. The OTUs that failed to align were excluded from downstream analysis.

10. Assign taxonomy using the Naïve Bayesian Classifier (RDP) implemented in Qiime (Wang *et al.*, 2007; Caporaso *et al.*, 2010b). We used Greengenes and SILVA databases for the 16S and 18S rDNA sequences respectively. In order to produce an RDP-friendly database for the 23S rDNA and the *tuf*A, we downloaded reference sequences from Genbank, used a phylogenetic similarity threshold (based on a UPGMA tree) to equalize the dataset (i.e. exclude repetitive species, which will bias the RDP classifier) and produced the reference dataset (one file with the sequences and another with taxonomic ranks). We used RDP taxonomic assignments to: i) infer the abundance of reads assigned to the main microbial groups (Figure 2); and ii) pre-filter OTUs to build a green algae phylogenetic tree: OTUs that were not classified as "Eukaryotic" (or "Chloroplast" in the 16S) were excluded from the phylogenetic analysis.

11. Filter OTUs found in negative controls (mock extractions and negative control PCRs). Although virtually no DNA was detected (with Qubit) in these controls, we added those samples to our library in order to detect any possible contaminant. But apart from cross contamination, sequencing errors can yield false-positives. Therefore some OTUs found in the controls could

be, for example, the most abundant *Ostreobium* sequences which should not be excluded. So we filtered the OTUs present in the controls, but only if they would represent less than 1% of the total number of reads. For the 18S dataset, OTUs matching Cnidaria and Dinophyceae were considered contaminants from the coral tissue and were also removed from the analysis.

12. Filter OTU table by minimum count (2) of reads per OTU per sample (filter_observations_by_sample.py - https://gist.github.com/adamrp/7591573). Another quality control step to remove OTUs present with low abundance in the samples.
13. Filter rare OTUs (less than 5 reads) and produce final filtered OTU fasta file. This is the input for the phylogenetic analysis.
14. Produce final OTU table and statistics. These statistics are another sort of quality check, and the OTU table is necessary for beta-diversity/comparative analysis (which we do not do in this study). From here one can check the sequencing depth and proceed to Qiime's core_diversity_analysis.py, for example.

Phylogenetic analysis:

The short reads generated by high-throughput sequencing technologies remain an issue for phylogenetic analysis. To overcome this problem, we used longer larger parts of the sequenced genes (available on Genbank, generated by Sanger sequencing) and additional genes to reconstruct the backbone of the green algal phylogeny. That way, even though the relationship within OTU-only clades may not be well resolved due to short fragments, the position of these clades among the green algae phylogeny can be inferred with strong support. We concatenated genes of different species of the same genus when same- species-sequences were not available, therefore filling as much as possible the gaps in the alignment. We used only species for which there was a reference sequence (for species or genus) for the marker analyzed – for example: there is no *Ostreobium* 18S rDNA sequence available (Supplementary Table 4), therefore this taxon was not included in the phylogenetic analysis of the 18S OTUs. The phylogenetic trees (Figure 3 and Supplementary Figures 1-4) were built with the following markers:

- *tuf*A-OTUs phylogeny: *tuf*A (including OTUs) + *rbcL* + 18S rDNA = 4833 bp.
- 16S rDNA-OTUs phylogeny: *tuf*A + *rbcL* + 18S rDNA + 16S rDNA (including OTUs) = 7251 bp.
- 18S rDNA-OTUs phylogeny: *tuf*A + *rbcL* + 18S rDNA (including OTUs) = 5297 bp.
- 23S rDNA-OTUs phylogeny: *tuf*A + *rbcL* + 18S rDNA + 23S rDNA (including OTUs) = 9489 bp.

Supplementary Table S4: Reference sequences (voucher and Genbank accession number, when available) used to reconstruct the green algae phylogeny.

Species	*tuf*A	*rbcL*	18S rDNA	16S rDNA	23S rDNA
Acetabularia acetabulum	DI1_HG518471	GB_HG518454.1	GB_AY165774		
Acetabularia dentata	HEC12349_XX	GB_AY177739.1	GB_Z33468		
Acrochaete leptochaete	MBLPoly1_AY454408	GB_AY303591			
Acrosiphonia sp.	GWS00746_HQ610			SAG127.80_FN563074.1	
Acutodesmus obliquus				UTEX.393_DQ396875	UTEX393_DQ396875
Auxenochlorella protothecoides				XX_KC843975	GB_KC843975
Avrainvillea lacerata	HV00599_FJ432651	GB_FJ432635.1		GB_FJ535833	
Avrainvillea nigricans	HV02664_XX	HV02664_XX		GB_FJ535834	
Batophora noID1	LL0095_XX				
Bolbocoleon piliferum	KMP01309a_KJ41191	GB_FJ715716.1	MA31b1_AY303599		
Boodleopsis sp.	H.0758_XX	H.0758_XX			
Botryococcus braunii				SAG.807.1_KM462884	SAG.807.1_KM462884
Bryopsis duplex	HV01238_XX				
Bryopsis hypnoides	GB_GQ892829	GB_GQ892829	GB_FJ715685	XX_GQ892829	GB_NC013359
Bryopsis plumosa	HV00880_XX	GB_AB038480	HV00880_FJ432630	West4718_LN810504	West4718_LN810504
Bryopsis vestita	F004_XX				
Callipsygma wilsonis	HV03983_XX	HV03983_XX	H.0890_XX		
Caulerpa filiformis	GB_CAD10730	GB_FR848349.1	GB_JF932262		
Caulerpa obscura	L.09.10.052_FR848335	GB_KF649941.1			
Caulerpa racemosa	GB_JN817682	GB_AB038485	GB_AF479702		
Caulerpa sertularioides					PGSTPM002_KF724396
Caulerpa simpliciuscula	L.09.10.048_FR848333	GB_FR848346.1			
Caulerpa verticillata	TS0072_KM186530	GB_FR668300.1	GB_JF932252		
Caulerpella ambigua	TS24_FJ432655	GB_FJ432638.1		GB_FJ535836	
Cephaleuros parasiticus	GB_AJM90125.1	GB_KM464711.1	GB_DQ399583		
Chlamydomonas reinhardtii	GB_DAA00908	GB_BK000554.2	GB_JN903984	GB_BK000554	GB_BK000554
Chlorella mirabilis				SAG.38.88_KM462865	SAG.38.88_KM462865
Chlorella sp.				ArM0029B_KF554427	ArM0029B_KF554427
Chlorella variabilis					NC64A_KJ718922
Chlorella vulgaris	C-27_BAA57886	GB_CHLC27	GB_X13688		
Chlorocladus australasicus	PH660_XX	GB_AY177750.1	GB_Z33466		
Chlorococcum oleofaciens		GB_KM052785.1	GB_U41176		
Chlorodesmis baculifera	H.0880_XX	H.0880_XX			
Chlorodesmis fastigiata				GB_FJ535837	
Chlorosarcina brevispinosa				UTEX1176_KM462875	UTEX1176_KM462875
Chlorosarcinopsis eremi	UTEX1186_HQ246369	BCPJT1VF80_HQ246349	BCPJT1VF8_HQ246315		
Choricystis sp.	CAUP.H.1984_XX	GB_KM438409.1	GB_AY762605	SAG.17.98_KM462878	SAG.17.98_KM462878
Cladophora pygmaea			GB_FM205051		
Cladophora rhodolithicola			GB_FM205053		
Cladophora socialis			GB_AB971263		
Cloniophora spicata	ARS00769_KM676565	ARS00515_KM677025			
Coccobotrys verrucariae		GB_AM260447.1	SAG1697_KM020110		
Coccomyxa sp.	C-169_HQ693844	GB_HQ693844.1		C.169_HQ693844	C-169_HQ693844
Codium arabicum.2	HV01432_XX	GB_EF107968.1			
Codium arenicola	HEC15968_KP685825	GB_JQ706329.1			
Codium bursa.2	G.113_XX	G.113_XX			
Codium duthieae	LT0248_JX463043	GB_EF107984.1	KZN2K4.1_FJ535848		
Codium edule					GB_EF426671.1
Codium fragile				GB_U08345.1	GWS002780_HQ603326.1
Codium sp.					GB_EF426672.1
Collinsiella tuberculata			WA3_AY198125		
Cymopolia barbata	XX_XX	XX_XX	HV00469_XX		
Dasycladus vermicularis	HV02661_XX	HV02661_XX			
Derbesia sp.	HADL602_XX				GWS008836_HQ603328.1
Desmochloris halophila				UTEX2073_HE610155	
Desmococcus endolithicus			GB_AJ431571		
Dicloster acuatus				SAG.41.98_KM462885	SAG.41.98_KM462885
Dictyochloropsis reticulata				SAG.2150_KM462860	SAG.2150_KM462860
Dunaliella salina	CCAP19-18_GQ250046	GB_GQ250046.1	GB_EF473745	CCAP.19.18_GQ250046	
Elliptochloris bilobata				CAUP.H7103_KM462887	CAUP.H7103_KM462887
Enteromorpha ovata	XX_KC66149				
Eremosphaera viridis	SAG.228.1_XX	GB_KM438414.1	GB_AF387154		
Ettlia pseudoalveolaris				UTEX975_KM462869	UTEX975_KM462869
Eugomontia sacculata			CCMP1673_AY198123		
Flabellia petiolata	HV01202_XX	GB_FJ432640.1	GB_AF416389	GB_FJ535847	
Floydiella terrestris	UTEX1709_ACZ58461	UTEX1709_NC014346	GB_D86498	UTEX1709_GU196268	UTEX1709_GU196268
Fusochloris perforata				SAG.28.85_KM462882	SAG.28.85_KM462882
Gayralia sp.	6969_JF680967				
Geminella minor				SAG.22.88_KM462883	SAG.22.88_KM462883
Geminella terricola				SAG.20.91_KM462881	SAG.20.91_KM462881
Gloeotilopsis sterilis	UTEX1704_KM462877	SAG888_KM020063	UTEX1704_KM462877	UTEX1704_KM462877	
Gomontia polyrhiza			MA24b1_AY278216		
Gonium pectorale				K3.F3.4_AP012494	K3-F3-4_AP012494
Halicoryne wrightii	HV00565_FJ535858	GB_AY177745.1	GB_AY165786		
Halicystis sp.	HV03191_XX	HV01207_XX			
Halimeda borneensis	HV00183b_AM049955	GB_FJ624514.1	PH534_AF525556		

Halimeda copiosa	H.0330_EF667065	GB_FJ624508.1	H.0330_AF525612		
Halimeda cryptica	HV00483_EF667057	GB_FJ624496.1	H.0237_AF407244		
Halimeda cylindracea	GB_AJF21965.1	GB_AJF21946.1	GB_AF525549.1		
Halimeda discoidea.ip	LPT0057_XX	GB_AB038488.1	SOC299_AF407254		
Halimeda fragilis.1	H.0125_EF667058	GB_FJ624498.1	H.0125_AF407245		
Halimeda micronesica	WLS184.0_EF667059	GB_FJ624499.1	H.0025_AF525578		
Halochlorococcum moorei	WA1.14B_AY454417		Wa14_AY198122		
Helicosp.oridium sp.				GB_DQ398104	GB_DQ398104
Hemichloris antarctica	UTEXEE124_KM464719.1	UTEXEE52_HQ317296.1	GB_FJ648517		
Ignatius tetrasporus				UTEX2012_FN563076	
Jaoa prasina	A_KM609	GB_JN102134.1	GB_JN102133		
Jenufa minuta			CAUPH8102_HM563744		
Koliella corcontica	SAG.24.84_XX	SAG.24.84_XX	GB_AJ306536	SAG.24.84_KM462874	SAG.24.84_KM462874
Koliella longiseta				UTEX339_KM462868	UTEX339_KM462868
Kornmannia leptoderma	GWS004830_HQ6105				
Leptosira terrestris	UTEX333_ABO69293	GB_EF506945.1	GB_Z28973	UTEX333_EF506945	UTEX333_EF506945
Lobosp.haera incisa	CAUP.H.4301_XX	CAUP.H.4301_XX	GB_AY762602	SAG.2007_KM462871	SAG.2007_KM462871
Marsupiomonas sp.				NIES1824_KM462870	NIES1824_KM462870
Marvania geminata				SAG.12.88_KM462888	SAG.12.88_KM462888
Micromonas sp.				RCC299_FJ858267	RCC299_FJ858267
Microthamnion kuetzingianum	CAUP.J.1201_XX	GB_KM438427.1	GB_AB488588	UTEX318_KM462876	UTEX318_KM462876
Monomastix sp.	OKE-1_ACK36861	GB_217314511_gb_FJ493 497.1	GB_FJ493496	OKE.1_FJ493497	OKE-1_FJ493497
Monostroma sp.	GWS003626_HQ610262	GWS003626_HQ603497	LYGJM_HQ850570		
Muriella zofingiensis	SAG21114_HQ902932	SAG21114_HQ902940			
Myrmecia israelensis	UTEX1181_723456786	UTEX1181_723456786	UTEX1181_KM462861	UTEX1181_KM462861	
Neocystis brevis				CAUP.D802_KM462873	CAUP.D802_KM462873
Nephroselmis astigmatica				NIES.252_KJ746600	NIES.252_KJ746600
Nephroselmis olivacea	NIES484_AF137379	NIES484_AF137379	GB_FN562436	NIES.484_AF137379	GB_AF137379
Ochlochaete hystrix	MA1.8d1_AY454406				
Oedogonium cardiacum	SAG575-1b_ACC97263	GB_EU677193.1	GB_U83133	SAG.575.1b_EU677193	GB_EU677193
Oltmannsiellopsis viridis	NIES360_ABB81968	GB_DQ291132.1	GB_FN562431	NIES.360_DQ291132	GB_DQ29113
Oocystis solitaria	SAG83.80_ACQ90812	GB_FJ968739.1	GB_AF228686	SAG.83.80_FJ968739	SAG.83.80_FJ968739
Ostreobiaceae sp.				GB_GU119643.1.1399	
Ostreobiaceae sp.2				GB_GU119844.1.1398	
Ostreobiaceae sp.3				GB_GU119848.1.1403	
Ostreobiaceae sp.4				GB_GU119621.1.1408	
Ostreobiaceae sp.5				GB_GU119562.1.1419	
Ostreobiaceae sp.6				GB_FJ203420.1.1418	
Ostreobiaceae sp.7				GB_FJ203501.1.1406	
Ostreobium quekettii	SAG699_XX	GB_AY004765.1		SAG699_XX	
Ostreobium sp.	H.0754_XX	GB_FJ535853.1			
Ostreococcus tauri	OTTH0595_CAL36350	GB_KC990831.1	GB_Y15841	OTTH0595_CR954199	OTTH0595_CR954199
Pabia signiensis				SAG.7.90_KM462866	SAG.7.90_KM462866
Parachlorella kessleri	SAG211-11g_ACQ90978	GB_FJ968741.1	GB_X56105	SAG.211.11g_FJ968741	SAG.211.11g_FJ968741
Paradoxia multiseta				SAG.18.84_KM462879	SAG.18.84_KM462879
Parvocaulis parvulus	HV01770_XX	GB_AY177741.1	GB_Z33471		
Pedinomonas minor	UTEXLB1350_ACQ90891	GB_FJ968740.1	GB_JN592588	UTEX.LB1350_FJ968740	UTEX-LB1350_FJ968740
Pedinomonas tuberculata					SAG.42.84_KM462867
Pedinophyceae sp.	YPF-701_AB561078				
Pedobesia simplex				GB_FJ535841.1.1486	
Penicillus capitatus	HV03128_XX	GB_FJ432641.1	H.0349_AF416404		
Percursaria percursa	UTEX143_AY454403	GB_AF387105.1	UTEX1423_AY303589		
Phaeophila dendroides	KMP01309b_KJ4119				
Picocystis salinarum	CCMP1897_AB561082	GB_AB491633.1	GB_FR865648	CCMP1897_KJ746599	CCMP1897_KJ746599
Planctonema lauterborni	SCCAP.K0187_XX	GB_KM438432.1	GB_AF387148	SAG.68.94_KM462880	SAG.68.94_KM462880
Pleodorina starri				NIES.1363_JX977846	NIES-1363_JX977846
Prasinococcus sp.	MBIC11011_AB561084	GB_AB491660.1	GB_AB058384	CCMP1194_KJ746597	CCMP1194_KJ746597
Prasinoderma coloniale				CCMP1220_KJ746598	CCMP1220_KJ746598
Prasinophyceae sp.	CCMP1205_AB561083	GB_AB491624.1	CCMP1205_U40921		CCMP1205_KJ746601
Prasiola crispa	P65_KF993457	GALW015724_JQ669724	GB_EF200532		
Prasiolopsis sp.	XX_XX	GB_KM464713.1	GB_AY762601	SAG.84.81_KM462862	SAG.84.81_KM462862
Protomonostroma undulatum	GWS00668_HQ61075	GB_HQ603507.1	18S_DQ821517		
Prototheca wickerhami				SAG.263.11_KJ001761	GB_KJ001761
Pseudendoclonium akinetum	UTEX1912_AAV80665	UTEX1912_AAV80617	GB_DQ011230	UTEX.1912_AY835431	UTEX1912_AY835431
Pseudochlorella pringsheimii	CAUP.H.1990_XX	GB_KM438438.1	GB_X63520		
Pseudochloris wilhelmii				SAG.1.80_KM462886	SAG.1.80_KM462886
Pseudochlorodesmis abbreviata	TS64_FJ432660	GB_FJ432649.1			
Pseudochlorodesmis sp.1	HV01204_FJ432656	GB_FJ432643.1	HV01204_XX		
Pseudococcomyxa simplex	CAUP.H.102_XX	GB_KM438443.1	GB_FN298926		
Pseudocodium devriesii	JH1_AM909695	GB_AM909690.1			
Pseudocodium floridanum	DML58944_FJ607676	GB_AM909692.1	NSF.I23_FJ432631		
Pseudocodium natalense	KZNb2250_FJ607679	GB_AM909693.1	KZNb2242_FJ432632		
Pseudoderbesia sp.	HV03339_LK0451				
Pseudomuriella engadinensis	UTEX58_HQ292755	UTEX58_HM770959	UTEX57_HM852441		
Pseudoneochloris marina	UTEX1445_AY4544				
Pterosp.erma cristatum	NIES626_AB561080	GB_U30281.1PCU30281	GB_AJ010407		
Pycnococcus provasolii	CCMP1203_ACK36845	GB_FJ493498.1	GB_X91264	CCMP.1203_FJ493498	GB_FJ493498
Pyramimonas parkeae	CCMP726_ACJ71140	GB_FJ493499.1	GB_FN562443	CCMP.726_FJ493499	GB_FJ493499
Rhipidosiphon javensis				GB_FJ535843	
Rhipilia coppejansii	PITI044_JQ082492	GB_JQ082482.1	G.453_XX		
Rhipilia crassa				GB_FJ535844	

Rhipilia nigrescens	HV00788_FJ432658	GB_FJ432646.1	HV00788_FJ432633		
Rhipiliopsis gracilis	HEC10439_XX	G.466_XX	HEC10439_XX		
Rhipiliopsis howensis	ASA001_XX	ASA001_XX			
Rhipiliopsis madagascariensis	TZ0333_XX	TZ0333_XX			
Rhipiliopsis peltata	H.0891_XX	H.0891.p2_XX	H.0891_XX		
Rhipiliopsis profunda	DML52138_XX	DML51973_FJ432647	DML51973_XX	GB_FJ535845	
Rhipiliopsis reticulata	DML67755_XX	DML68726_XX	GB_AF416386		
Rhipiliopsis stri	DML68740_XX	DML68740_XX			
Rhipocephalus phoenix				GB_FJ535846	
Ruthnielsenia tenuis	RNtenuis7_JQ30995				
Scenedesmus obliquus	UTEX393_ABD48278	UTEX393_ABD48257	GB_AJ249515		
Schizomeris leibleinii	UTEX-LB1288_HQ700713	UTEX-LB1288_HQ700713	GB_AF182820	UTEX.LB1228_HQ700713	UTEX.LB1228_HQ700713
Spongomorpha aeruginosa	GWS003854_HQ61078				
Stichococcus sp.	CAUP.J.1302_XX	GB_KM438447.1	GB_DQ275461	UTEX176_KM462864	UTEX176_KM462864
Stigeoclonium helveticum	UTEX441_ABF60202	GB_DQ630521.1	GB_U83131	UTEX.441_DQ630521	UTEX441_DQ630521
Tetracystis aeria		GB_EF113476.1	GB_U41175		
Tetraselmis sp.	NIES2432_AB561081		GB_U05039		
Trebouxia aggregata	SAG219-1d_EU123976	GB_EU123967.1	GB_EU123942		
Trebouxiophyceae sp.	MX-AZ01_JX402620	GB_JX402620.1		MX.AZ01_JX402620	
Trentepohlia annulata	GB_KM464717	GB_KM464712.1	SAG20.94_ KM020077		
Trochiscia hystrix		GB_EF113480.1	SAG10380_KM020157		
Tydemania expeditionis	FL1151_LN810505	FL1151_LN810505	HV00873_FJ432634	FL1151_LN810505	FL1151_LN810505
Udotea flabellum	HV02674_XX	HV02674_XX	H.0415_AF407270		
Ulothrix zonata	UTEX745_AY45444	GB_AF499683.1	112011_JX491158		
Ulva arasakii	GB_AB561079	GB_AB097621.1	GB_AJ000040		
Ulva fasciata	GWS023543_JN029306	GB_AY422565	GB_DQ286547		
Ulva shanxiensis	SAS.06035_KJ617036				
Ulva sp.				UNA00071828_KP720616	UNA00071828_KP720616
Ulvaria sp.	GWS01793_KM55060				
Ulvella endozoica	UTEXB351_JQ30993				
Ulvella sp.	B_KM608	HB1306_KM226205	HB1306_KM226210		
Ulvella waernii	RN18981_JQ3099				
Umbraulva japonica	GWS018246_JN029346	C14_AB426255			
Umbraulva sp.	GWS03894_JN09359	GB_AB097612.1			
Urosp.ora wormskioldii	GWS006374_HQ610441	GWS006374_HQ603676	Park1_AY476819		
Volvox carteri	UTEX2908_ACY06012	GB_EU755275.1	GB_AB542923		
Watanabea reniformis	CAUP.H.1932_XX	GB_KM464714.1	GB_X73991	SAG.211.9b_KM462863	SAG.211.9b_KM462863
Xylochloris irregularis	CAUP.H.7801_XX	GB_KM438451.1	GB_EU105209	CAUP.H7801_KM462872	CAUP.H7801_KM462872

References:

Caporaso J, Kuczynski J, Stombaugh J, Bittinger K, Bushman FD, Costello EK, *et al.* (2010). QIIME allows analysis of high-throughput community sequencing data. *Nat Methods* **7**: 335–336.

Caporaso JG, Bittinger K, Bushman FD, Desantis TZ, Andersen GL, Knight R. (2010). PyNAST: A flexible tool for aligning sequences to a template alignment. *Bioinformatics* **26**: 266–267.

Caporaso JG, Lauber CL, Walters WA, Berg-Lyons D, Huntley J, Fierer N, *et al.* (2012). Ultra-high- throughput microbial community analysis on the Illumina HiSeq and MiSeq platforms. *ISME J* **6**: 1621–1624.

Cremen C, Huisman JM, Marcelino VR, Verbruggen H. (2016). Taxonomic revision of *Halimeda* in southwestern Australia. Aust Syst Bot. **29**: 41-54

Edgar RC. (2013). UPARSE: highly accurate OTU sequences from microbial amplicon reads. *Nat Methods* **647**: 1–5.

Fama P, Wysor B, Kooistra WHCF, Zuccarello GC. (2002). Molecular phylogeny of the genus *Caulerpa* (Caulerpales, Chlorophyta) inferred from chloroplast *tuf*A gene. *J Phycol* **38**: 1040–1050.

Gilbert JA, Jansson JK, Knight R. (2014). The Earth Microbiome project: successes and aspirations. *BMC Biol* **12**: 69.

Katoh K, Misawa K, Kuma K, Miyata T. (2002). MAFFT: a novel method for rapid multiple sequence alignment based on fast Fourier transform. *Nucleic Acids Res* **30**: 3059–3066.

Klindworth A, Pruesse E, Schweer T, Peplies J, Quast C, Horn M, *et al.* (2013). Evaluation of general 16S ribosomal RNA gene PCR primers for classical and next-generation sequencing-based diversity studies. *Nucleic Acids Res* **41**: e1.

Magoč T, Salzberg SL. (2011). FLASH: Fast length adjustment of short reads to improve genome assemblies. *Bioinformatics* **27**: 2957–2963.

Porazinska DL, Giblin-Davis RM, Faller L, Farmerie W, Kanzaki N, Morris K, *et al.* (2009). Evaluating high-throughput sequencing as a method for metagenomic analysis of nematode diversity. *Mol Ecol Resour* **9**: 1439–1450.

Rohland N, Reich D. (2012). Cost-effective, high-throughput DNA sequencing libraries for multiplexed target capture. *Genome Res* **22**: 939–46.

Schmieder R, Edwards R. (2011). Quality control and preprocessing of metagenomic datasets. *Bioinformatics* **27**: 863–864.

Sherwood AR, Presting GG. (2007). Universal primers amplify a 23S rDNA plastid marker in eukaryotic algae and cyanobacteria. *J Phycol* **43**: 605–608.

Steven B, McCann S, Ward NL. (2012). Pyrosequencing of plastid 23S rRNA genes reveals diverse and dynamic cyanobacterial and algal populations in two eutrophic lakes. *FEMS Microbiol Ecol* **82**: 607–615.

Wang Q, Garrity GM, Tiedje JM, Cole JR. (2007). Naïve Bayesian classifier for rapid assignment of rRNA sequences into the new bacterial taxonomy. *Appl Environ Microbiol* **73**: 5261–5267.

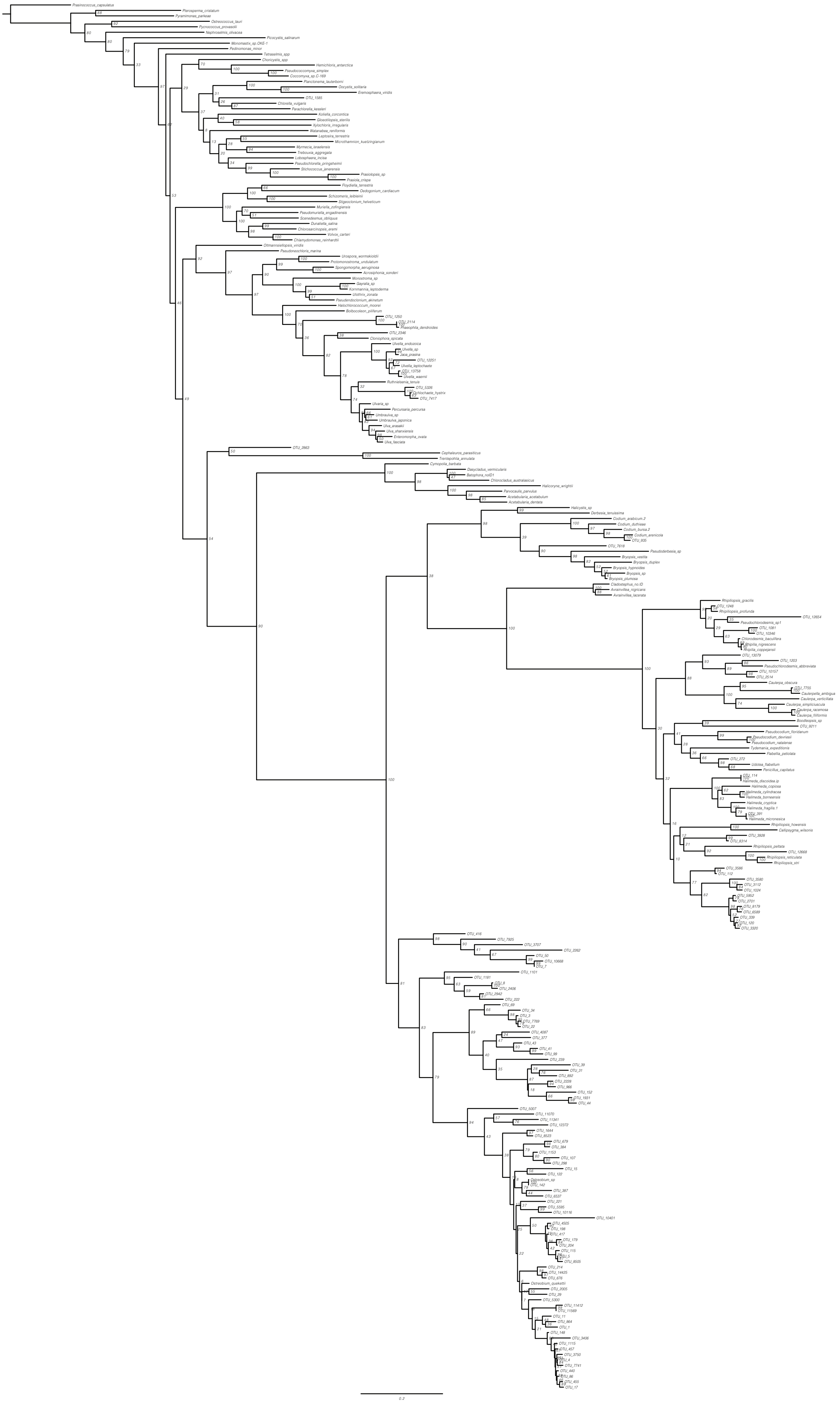

Supplementary Figure S1 – Maximum Likelihood phylogeny including *tuf*A OTUs with bootstrap values.

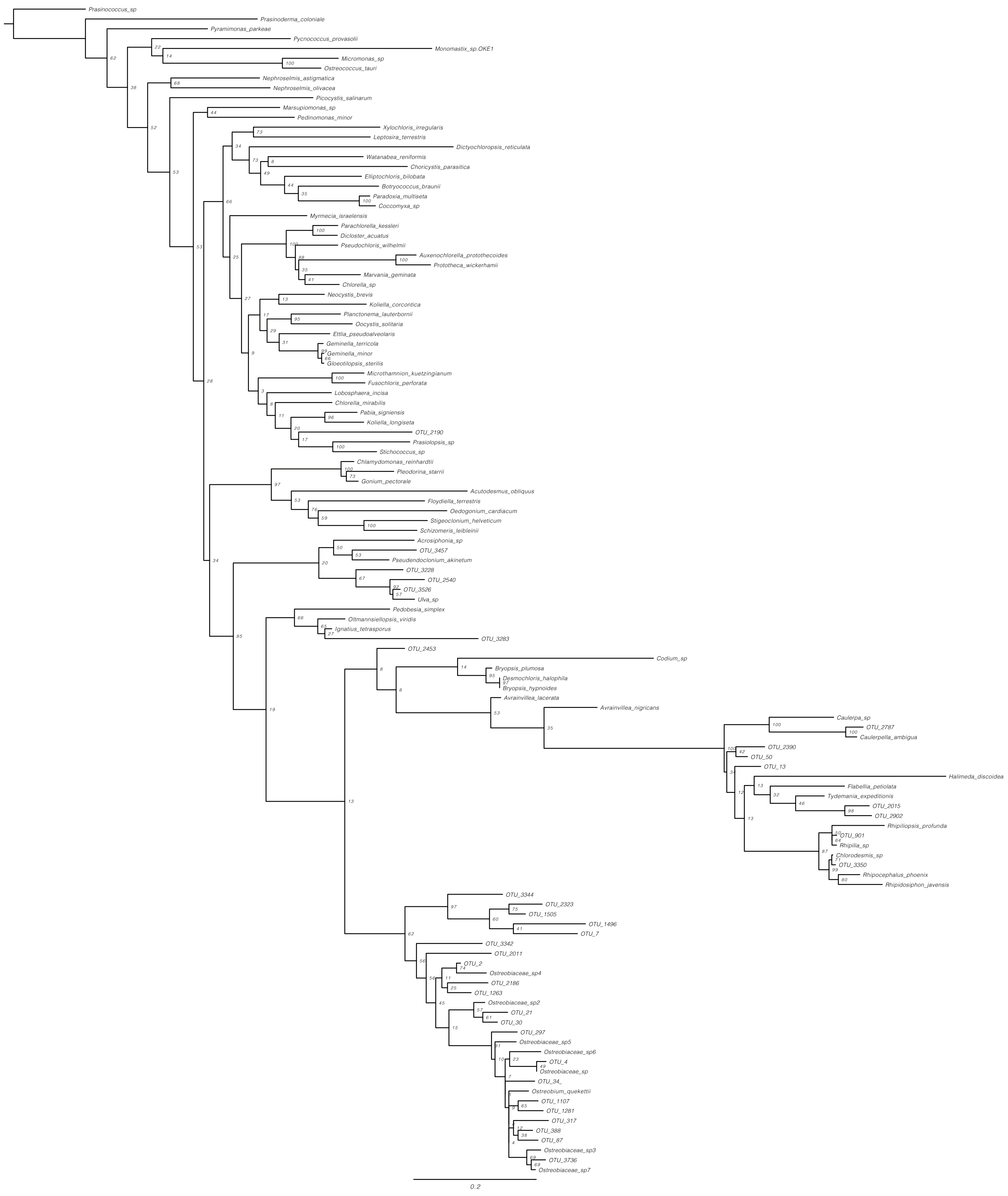

Supplementary Figure S2 – Maximum Likelihood phylogeny including 16S rDNA OTUs with bootstrap values.

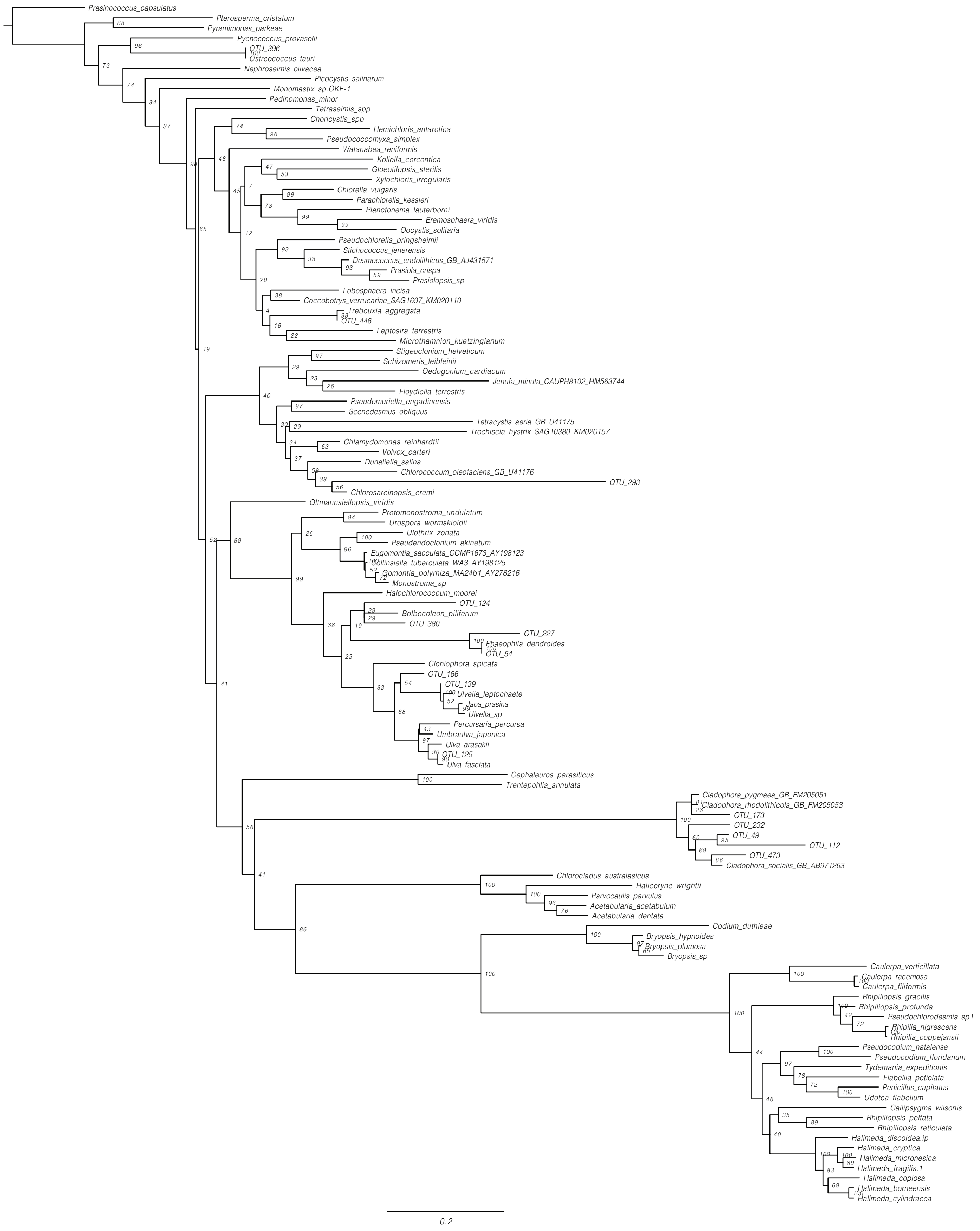

Supplementary Figure S3 – Maximum Likelihood phylogeny including 18S rDNA OTUs with bootstrap values

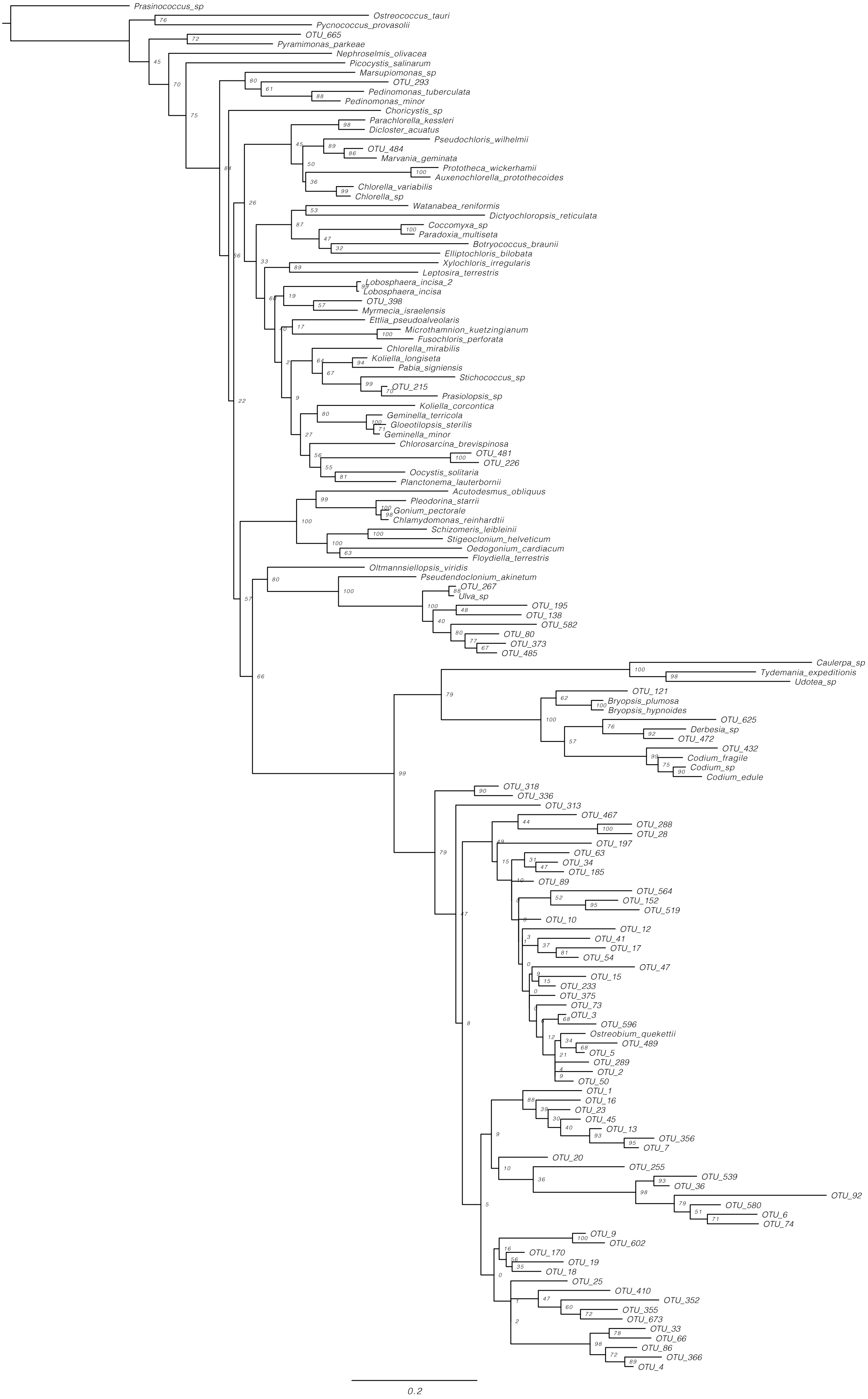

Supplementary Figure S4 – Maximum Likelihood phylogeny including 23S rDNA OTUs with bootstrap values
